# Adaptive evolution of loci covarying with the human African Pygmy phenotype

**DOI:** 10.1007/s00439-012-1157-3

**Published:** 2012-03-11

**Authors:** Isabel Mendizabal, Urko M. Marigorta, Oscar Lao, David Comas

**Affiliations:** 1 Departament de Ciències de la Salut i de la Vida, Institut de Biologia Evolutiva (CSIC-UPF), Universitat Pompeu Fabra, 08003 Barcelona, Spain; 2 Department of Forensic Molecular Biology, Erasmus MC University Medical Center Rotterdam, 3015 GE Rotterdam, The Netherlands

## Abstract

African Pygmies are hunter-gatherer populations from the equatorial rainforest that present the lowest height averages among humans. The biological basis and the putative adaptive role of the short stature of Pygmy populations has been one of the most intriguing topics for human biologists in the last century, which still remains elusive. Worldwide convergent evolution of the Pygmy size suggests the presence of strong selective pressures on the phenotype. We developed a novel approach to survey the genetic architecture of phenotypes and applied it to study the genomic covariation between allele frequencies and height measurements among Pygmy and non-Pygmy populations. Among the regions that were most associated with the phenotype, we identified a significant excess of genes with pivotal roles in bone homeostasis, such as *PPPT3B* and the height associated *SUPT3H*-*RUNX2*. We hypothesize that skeletal remodeling could be a key biological process underlying the Pygmy phenotype. In addition, we showed that these regions have most likely evolved under positive selection. These results constitute the first genetic hint of adaptive evolution in the African Pygmy phenotype, which is consistent with the independent emergence of the Pygmy height in other continents with similar environments.

**Electronic supplementary material:**

The online version of this article (doi:10.1007/s00439-012-1157-3) contains supplementary material, which is available to authorized users.

## Introduction

African Pygmy groups are defined by phenotypic, geographic and cultural characteristics (Cavalli-Sforza 1986). Among these, the most relevant are their short stature (hereinafter referred to as the Pygmy phenotype as in Perry and Dominy (2009)), representing one extreme of the height spectrum in humans, and their lifestyle as hunter gatherers, at least until recently, in the African rainforest (Cavalli-Sforza 1986). The Eastern (Democratic Republic of Congo and Rwanda) and Western Pygmies (Cameroon, Republic of Congo, Gabon and the Central African Republic) represent the two principal groups of African Pygmies.

Strong evidence supports that Pygmy height is genetically determined (Becker et al. 2011) and more specifically, that the genetic architecture of the African Pygmy phenotype is polygenic and additive. First, the offspring of Pygmy/non-Pygmy intermarriages present intermediate statures (Cavalli-Sforza 1986). Secondly, Mbuti Pygmies (Eastern Pygmies) not only represent the shortest Pygmy group, but also show the lowest admixture rates with non-Pygmy populations in Africa (Patin et al. 2009). In contrast, the so-called Pygmoid populations, characterized by being considerably taller than Pygmies, show evidence of extensive admixture with neighboring farmer populations (Patin et al. 2009; Verdu et al. 2009). Thirdly, the farmer populations living near the forests inhabited by Pygmies also show smaller stature than other farmers that have no contact with Pygmy groups (Cavalli-Sforza 1986). To date, biochemical alterations have been reported in the growth hormone-insulin-like growth factor axis (*GH*-*IGF*) in African Pygmy populations (Bozzola et al. 2009; Jain et al. 1998; Merimee et al. 1981; Rimoin et al. 1969). However, the genetic basis and pathways involved in this phenotype remain elusive.

So far, there is no definitive evidence supporting adaptation for small body size in humans. Accordingly, the putative adaptive advantage of the Pygmy phenotype is still under debate (Perry and Dominy 2009). Different hypotheses have been proposed to explain a higher fitness associated with the Pygmy stature: thermoregulatory adaptation to the rainforest (Cavalli-Sforza 1986), adaptation to a food-limited environment (Hart and Hart 1986), improvement of mobility in the dense tropical forest (Diamond 1991), and adaptation for an earlier reproductive age due to shortened lifespan (Migliano et al. 2007). Cavalli-Sforza (1986) hypothesized that both the Pygmy phenotype and the “long-elongated African phenotype” (such as the Maasai body shape) may represent two extremes of adaptation to two extremely hot climates: uniformly hot wet (in the case of the rainforest) and hot dry (in the case of the savanna). In any case, the fact that the Pygmy phenotype has evolved at least three times independently in human rainforest populations in Africa, South America and South-east Asia supports a major role of adaptation (Perry and Dominy 2009).

A large number of loci have been described to contribute to height in European and Asian populations, but these typically explain small fractions of phenotypic variation (Lango Allen et al. 2010). The large population differences in height observed between Pygmy and non-Pygmy populations (30 cm in average between the Maasai and Mbuti) and the putative role of natural selection in its evolution suggests that the architecture of the Pygmy stature could be different from that of normal height variation. Specifically, the architecture of such a differentiated and putatively adaptive phenotype could be due to a limited set of variants with moderate to large phenotypic effects. Therefore, whereas association studies of non-Pygmy height variation demand very large sample sizes, a population-based study with small sample sizes could be justified for the Pygmy height.

The objective of the present work is to analyze dense genome-wide SNP data from Pygmy and non-Pygmy populations (Altshuler et al. 2010; Li et al. 2008) to gain insights into the genetic architecture of the Pygmy phenotype and the putative adaptive evolutionary forces that shaped it. Since the trait is not shared by neighboring non-Pygmy populations, we hypothesize that (1) the genomic regions responsible for this trait must show high differentiation between Pygmy and non-Pygmy groups (2) the analysis of linked tagSNPs allows the identification of these highly differentiated regions, and (3) the genetic loci responsible for the phenotype may covary with height at a population level. By developing new statistics that measure population genetic and phenotypic differentiation, we provide a list of regions that show strong population association with the Pygmy phenotype, including height associated genes involved in bone remodeling. Finally, we propose that adaptation is most likely involved in the evolution of the loci that covary with the Pygmy phenotype.

## Materials and methods

### Data

The genetic data analyzed in this study consisted of genotypes generated on Illumina 650Y platform on the HGDP-CEPH panel (Li et al. 2008) and HapMap Phase III (Altshuler et al. 2010). The HGDP-CEPH panel dataset consisted of 21 Biaka Pygmy individuals, 13 Mbuti Pygmies, 21 Yorubans and 28 French individuals from the standardized subset (Rosenberg 2006). A total of 644,237 autosomal SNPs for which genotypic information was available for all four populations were considered. Samples from HapMap Phase III (Altshuler et al. 2010) considered were: 82 Luhya (Bantu) individuals from Webuye (Kenya), 107 Yoruba from Ibadan (Nigeria) and 139 Maasai from Kinyawa (Kenya). A total of 471,785 SNPs overlapped between HapMap and the Illumina 650Y dataset after removing the non-autosomal SNPs. Phenotypic data was recovered from the literature and consisted of mean and standard deviations of height in adult males within the Mbuti, Biaka, Yoruba, Luhya and Maasai populations (Table S1 in Supplementary Material).

### Estimation of population association between height and SNP variation within African populations (CI_*n*_)

We aimed to identify markers that covary with height at a population level among the different populations. We derived a new statistic based on *I*_*n*_ (Rosenberg et al. 2003), under the assumption that the genetic basis of height were shared by both Pygmy groups and other African populations. The statistic measures how much extra information the phenotype (*P*) contains to infer the geographic origin of one individual (*Q*) after removing the information that is provided by knowing its genotype (*J*) (Supplementary Material). For instance, knowing an individual is 140 cm tall increases the probability that this individual belongs to a Pygmy population rather than to the Maasai. With the aim of identifying the causal genetic variants of the phenotype, one would seek in the genome for SNPs that provide as much information as that contained in the phenotype to assign that individual to each of the populations. Specifically, the conditional informativeness (CI_*n*_) of the phenotype over genotype is

$$ {\text{CI}}_{n} = I_{n} (Q;P|J) = I_{n} (Q;P;J) - I_{n} (Q;J) $$

The closer the CI_*n*_ is to 0 the more similar the amount of information gathered from allele frequencies is compared to that of considering both allelic frequencies and height information. Therefore, markers showing the lowest CI_*n*_ values are those that covary most strongly with the phenotypic variation in the considered populations. Using the genomic distribution of this statistic, we can identify the loci that compared with the rest of the genome covary most with the phenotype and thus are most likely to have phenotypic effects on the trait. Therefore, the power to identify trait-associated markers will depend on two factors: (1) how well the genomic background associates with the trait (i.e. decreased power if the demographic history follows the same cline as the phenotype) and (2) the amount of genetic differentiation at the causal loci as compared to the rest of the genome. For instance, our statistic should perform well under a scenario of positive selection, as adaptive pressures on a phenotype would create large differences in allele frequencies in causal genes, standing out from the average genomic differentiation. We studied the association of allele frequencies of each SNP with the population distributions of height in five populations: Eastern and Western Pygmies (Mbuti and Biaka, respectively) as well as in three non-Pygmy Africans (including Yoruba, Luhya and Maasai) (Table S1 in Supplementary Material). We specifically excluded non-African populations to avoid the stronger secular trends in height in regions with post-industrial economies (Silventoinen 2003).

### Assessing the power of conditional *I*_*n*_ statistic

Since the power of the statistic will depend on the background noise due to genetic substructure, we first evaluated the percentage of markers in the genome that followed the phenotypic cline. Specifically, for each SNP we ordered the populations according to the minor allele frequencies, and compared with the order of populations according to height measurements (Mbuti ≤ Biaka < Yoruba/Luhya < Maasai). We observed that the most common order in the genome follows the phenotypic trait (Figure S1 in Supplementary Material). However, it is also noticeable that the demographic history of these five populations has been complex, since only 15% of the markers follow the cited pattern, whereas the remaining 85% is subdivided in other clines. The inclusion of several populations with different demographic histories is expected to reduce the number of markers that covary with the phenotype due to random processes, thus restricting the effect of population substructure on the rate of false positives.

We explored the phenotypic outcome of 192 different genetic architectures, ranging from 3 to 1,000 causal SNPs with phenotypic effects ranging from 10 to 0.01 cm per allele (Table S2 in Supplementary Material). By choosing random SNPs from the genome and considering them as causal, we used their allele frequencies to create phenotypic distributions for each population under an additive model. To obtain realistic phenotypes, we considered that the causal SNPs followed this minor allele frequency order: Mbuti ≤ Biaka < Yoruba/Luhya < Maasai. At this step, we considered two different cases (scenarios 1, 2). For the first scenario we chose random SNPs from the genome with the single condition of the cited minor allele frequency order. For the second scenario, we additionally forced that the causal SNPs would show a minimum difference of 0.37 (95% genomic quantile) in allele frequency between Maasai and Mbuti. Then, we calculated the phenotypic distributions for each architecture and chose the four most realistic ones regarding the ~30 cm height difference between Maasai and Mbuti populations. The reason why we focused on the difference between these two groups was because we aimed to find the genetic basis of the differences in height between Pygmy and non-Pygmy populations, instead of simulating the human height itself (i.e. the basal 140 cm in all human populations).

We also explored two additional cases considering that the phenotype does not follow a common demographic cline (scenario 3) and that the causal SNPs show high differentiation (scenario 4). When computing the statistic for the third and forth scenarios, we used the same causal SNPs as in scenarios 1 and 2, respectively but changed the population labels so that the allele frequency order was a rare one in the genome (see Figure S1 in Supplementary Material). For each selected architecture, we ran 10 independent replicates taking the causal SNPs from the genome, generating the phenotypic distributions and computing CI_*n*_ with them. To compute the power of the statistic, we averaged the number of times the real causal variants were in the 5% extreme of the empirical distribution over the replicates. Owing to computational reasons, we ran these simulations with a random subset of 20,000 SNPs from the genome which did not statistically differ from the values of the statistic in the whole dataset.

### Population genetic differentiation between Pygmy and non-Pygmy populations (PI_*n*_)

**Fig. 1:**
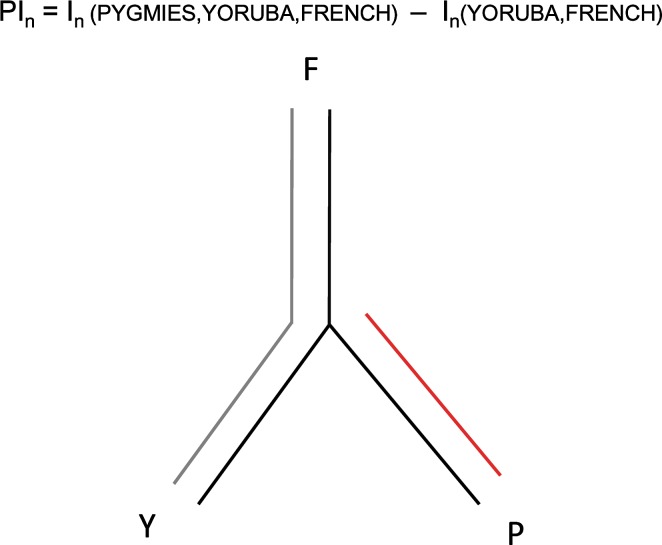
Pygmy-specific informativeness for assignment (PI_*n*_). The Pygmy-specific informativeness for assignment statistic (represented by the *red branch*) is computed as the subtraction of the informativeness for assignment statistic computed on the non-Pygmy populations (represented by the *gray branches*) to the grouping of all three populations (represented by the *black tree*). This subtraction allows the distinction between markers that differentiate Pygmy versus non-Pygmy populations. *F* French, *Y* Yoruba, *P* Pygmies (color figure online)

The main limitation of the CI_*n*_ statistic is that it assumes that the genetic loci responsible for height are shared in the five African populations. This assumption would not hold if the causal loci were Pygmy specific and therefore absent in other African non-Pygmy populations. To identify the genomic regions that show highest genetic differentiation between Pygmy and non-Pygmy populations, we computed the informativeness for assignment (*I*_*n*_) for each marker (Rosenberg et al. 2003). To ascertain markers that are informative to differentiate Pygmy populations, we computed two different estimates of the *I*_*n*_ statistic. First, we calculated the *I*_*n*_ for the grouping of three populations (1) Pygmy (either Biaka, Mbuti or both) (2) Yoruba and (3) French. Subsequently, we subtracted the *I*_*n*_ computed using only French and Yoruba. Hence, PI_*n*_ = *I*_*n*_ (Yoruba, French, Pygmy) − *I*_*n*_ (Yoruba, French). By means of this subtraction, we were able to distinguish between Pygmy versus non-Pygmy differentiation (Fig. 1), an approach that is similar to the locus-specific branch lengths (LSBLs) (Shriver et al. 2004). For instance, if the Pygmy to non-Pygmy differentiation were absolute, i.e. for a given SNP, the Pygmy population had one allele fixed, whereas the Yoruba and French showed the alternative allele fixed, the PI_*n*_ statistic would reach the maximum value around ln(2) ~0.63 (as 2 are the maximum number of populations that may be distinguished by means of a bi-allelic marker; Rosenberg et al. 2003). On the other hand, if the differentiation of the three populations were due to either Yorubans or French (and if Pygmy completely shared the same allele frequencies with 1 of the 2 populations), the PI_*n*_ value would be negative. Therefore, markers with large positive PI_*n*_ values are of interest to distinguish differentiation specific to the Pygmy branch. To test whether the choice and number of the non-Pygmy populations (Yoruba and French) could bias the PI_*n*_ results, we repeated the analysis with a different set of populations covering Africa and Eurasia (Mbuti, Biaka, Mandenka, Yoruba, Kenyan Bantu, French, Russian and Han, taken from the standardized subset of individuals on the same SNP dataset; Li et al. 2008; Rosenberg 2006). A high correlation between the two datasets showed that the statistic computed on the Yoruba and French captured the targeted Pygmy-specific differentiation (Pearson’s correlation coefficient *r* = 0.83 between empirical *p* values of both datasets).

### Study of the genomic distribution of the statistics

To keep the number of false positives as low as possible, we investigated the spatial distribution in the genome of the previously described statistics (PI_*n*_ and CI_*n*_) by means of local Moran’s *I* statistic (Anselin 1995) computed for each SNP. The rationale is that some markers are expected to spuriously show strong departures of the statistic of interest, whereas the study of several markers in the same genomic region should be more robust to these random events, and more likely to reflect real demographic and/or selective histories. In addition, it permits to survey non-genotyped causal variants, provided that they are in linkage with markers present in the genotyping panel. We defined a surrounding 200 Kb window for each marker, and the SNPs within this window were assigned a weight of 1, whereas markers elsewhere in the genome had a weight of 0. Markers showing high-positive values of local Moran’s *I* (positive spatial autocorrelation) indicate that the core SNP is located in a region in which nearby markers have similar values of the statistic in comparison to the genomic background value. Thus, we aimed to detect clusters of highly differentiated (PI_*n*_) or height covarying (CI_*n*_) SNPs. In the case of PI_*n*_, we subsequently excluded those markers showing negative PI_*n*_ values since these represent differentiation in French and/or Yorubans. After the normalization of the local Moran’s *I* values, we obtained the local Moran’s *I* distributions, which under the null hypothesis of no spatial autocorrelation are normal distributions with *μ* = 0 and *σ* = 1 (Anselin 1995). We obtained the significance of the spatial autocorrelation distributions by setting a *p* value cut off (one tailed, *α* = 0.05) and correcting for multiple testing by Bonferroni (*p* values of the significant SNPs are reported in Table S3 in Supplementary Material). Whenever two significant markers were located within the same 200 Kb window, we considered they belonged to the same genomic regions. We checked the overlap of these regions with previous selection scans based on extended haplotype tests performed on both African Pygmies (López Herráez et al. 2009) by a binomial test, dividing the genome in windows of 200 Kb.

### Demographic simulations

We performed simulations under the most plausible demographic scenarios and assessed the feasibility of detecting loci under selection by studying significant departures of our statistics. We first estimated the demographic parameters by approximate Bayesian computation (ABC) (see Supplemental Methods and Figure S2 in Supplementary Material). Subsequently, we drew 1,000 values from the posterior distributions of each parameter (Table S4 in Supplementary Material) and used them to simulate 1,000 different genome stretches of 5 Mb with Cosi simulator (Schaffner et al. 2005). To make the simulated and real datasets comparable regarding the known ascertainment bias in genotyping arrays, we selected the SNPs from the simulations to fulfill different aspects of the Illumina dataset including (1) density of 1 SNP per 5 Kb (2) SNP spacing (3) MAF spectrum and (4) LD block length (Figure S2 in Supplementary Material).

### Enrichment of genic and non-synonymous variants

The functional categories for the SNPs were retrieved from Ensembl 18, Homo sapiens variation 58, “consequence to transcript” attribute. The “non-synonymous”, “synonymous”, “3′UTR” and “5′UTR” SNPs were considered genic, whereas the “intergenic” SNPs were considered nongenic. We checked the proportion of genic and non-synonymous (NS) SNPs versus the nongenic ones in the tails of the spatial autocorrelation in PI_*n*_ and CI_*n*_. The statistical significance was tested by bootstrapping the number of markers found in the cutoff of the distribution for each replicate. Based on the 1,000 bootstrapping replicates, we computed the percentage of times that the ratio obtained was smaller than the observed value.

### Functional pathway analysis

We subjected the candidate genes detected in our study to the Ingenuity pathway analysis (http://www.ingenuity.com). The analysis is based on the Ingenuity Pathways Knowledge Base repository collected from the full text of the peer-reviewed literature in life sciences. The functional relationships considered in the database were only those experimentally confirmed. Fisher’s exact test was used to calculate the *p* value determining the probability that the association between the genes in the dataset and the canonical pathway was explained by chance alone.

## Results

### Power of conditional Informativeness to detect genomic covariation with height

We first validated the CI_*n*_ statistic to detect the genomic region containing the variant associated with a phenotype with a monogenic trait, the lactase persistence in Europe, one of the best known examples of positive selection in humans. The trait is explained by a north–south cline in frequency of the T/C polymorphism at position −13,910 upstream the *LCT* gene (Enattah et al. 2002). We applied our method by incorporating the phenotypic information, that is, the percentage of lactose tolerant adults in three populations of Northern, Central and Southern European ancestry (Itan et al. 2010). Our method performed exceptionally well in distinguishing the *LCT* gene in chromosome 2 as the unique genome-wide significant signal found (Figure S1 in Supplementary Material).

**Fig. 2:**
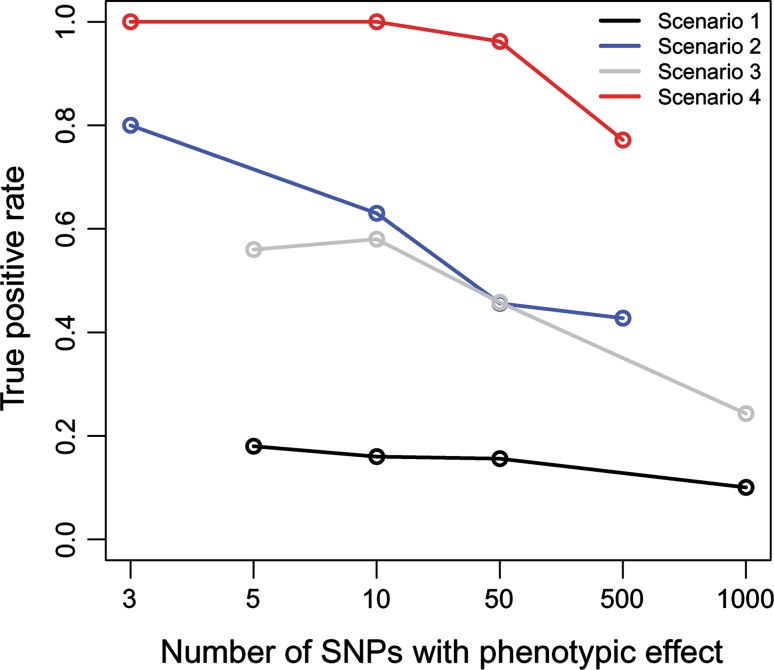
Power analysis of the CI_*n*_ statistic for the Pygmy phenotype. The plot shows the rate of true positives for each of the simulated scenarios with the following additive genetic architectures considered: scenarios 1 and 3: 5 SNPs with 10 cm effect per allele copy, 10 SNPs with 5 cm, 50 SNPs with 1 cm and 1,000 SNPs with 0.05 cm each; scenarios 2 and 4: 3 SNPs with 10 cm, 10 SNPs with 3 cm, 50 SNPs with 0.7 cm and 500 SNPs with 0.07 cm

Next, we aimed to validate the statistic for polygenic traits. When considering the current limitations to simulate polygenic phenotypes under different evolutionary contexts, we assessed the power of our statistic by studying the genomic distribution of CI_*n*_ for the Pygmy phenotype. Specifically, we analyzed four different scenarios (see “Materials and methods” for a full description): scenario 1 considered that the phenotype was produced by markers following the most common demographic cline, whereas scenario 2 considered that the phenotype was produced by highly genetically differentiated markers following that demographic cline. In contrast, scenario 3 assumed that the phenotype was due to markers that followed a rare demographic cline and scenario 4 further included strong differentiation in the causal markers. As expected, for the four scenarios the power decreased as the number of causal SNPs increased and the corresponding phenotypic effect decreased (Fig. 2). For the first scenario, the statistic showed limited power (around 20%) for the Pygmy phenotype in the considered populations, suggesting that if the SNPs responsible for the phenotype did not stand out from the background genomic allele frequencies, we would have little chances to capture most of the real phenotypic variants. Nevertheless, if the causal genomic regions showed high genetic differentiation, the power to detect many of them would be high, around 80% if there were three causal SNPs or above 50% if <500 SNPs were involved. In the third scenario (the hypothetical case of the phenotype not following the demographic cline), we observed that even though causal SNPs showed low differentiation (close to the genomic average), the power of the statistic was considerable (above 50% if <50 SNPs). Finally, in the fourth scenario (the phenotype did not follow the demographic cline and the causal SNPs showed high differentiation) the power of the statistic was above 75% even considering a polygenic trait with 500 causal SNPs. As the Pygmy phenotype follows (at least partially) the demographic cline (Figure S1 in Supplementary Material) scenarios 1 or 2 would be more realistic. Therefore, if the African Pygmy phenotype is largely determined by less than a few hundred of SNPs that show large population genetic differentiation, we expect that the extremes of the CI_*n*_ distributions will be enriched for true loci involved in the Pygmy stature.

### Candidate regions for the Pygmy phenotype

**Fig. 3:**
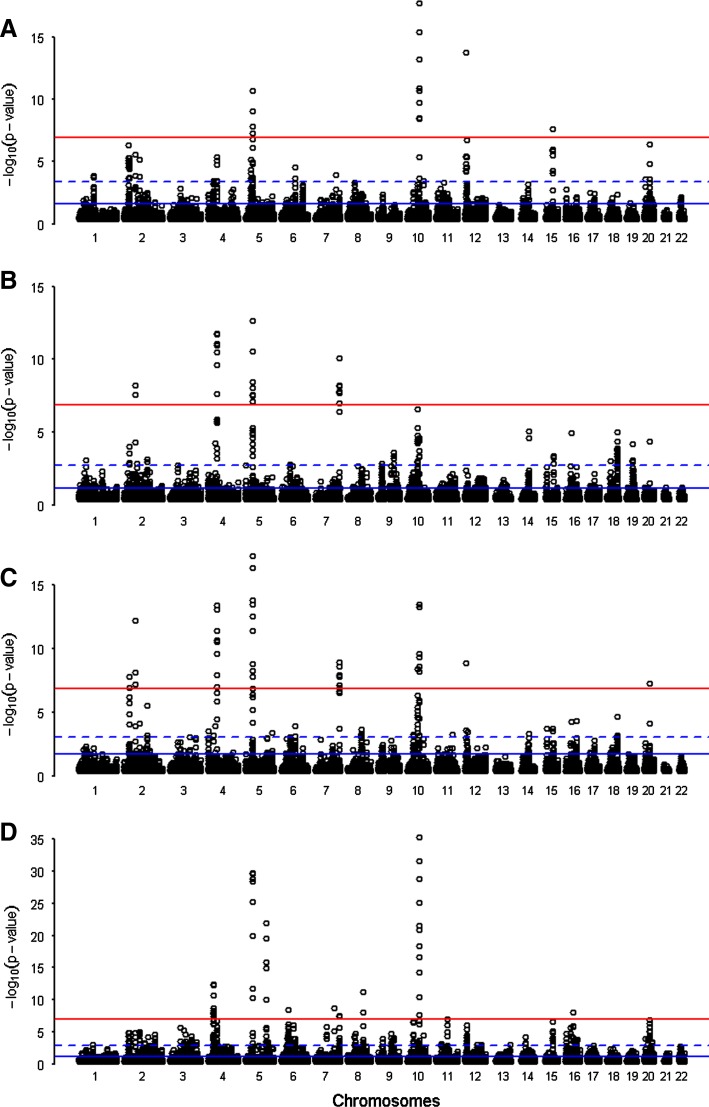
Manhattan plots showing the results for spatial autocorrelation distributions in PI_*n*_ and CI_*n*_ statistics. Plots showing results for the spatial autocorrelation in PI_*n*_ (**a** Mbuti, **b** Biaka and **c** Pygmies grouped together) and CI_*n*_ (**d**) statistics in the autosomal chromosomes. *Red lines* indicate the significance threshold applied in the observed dataset, whereas the *blue lines* indicate the spatial autocorrelation values found in 1,000 demographic simulations (*dashed lines* mark the maximum value, whereas *solid lines* indicate 99% quantile of distributions) (color figure online)

We identified the outlier SNPs for the spatial autocorrelation values of CI_*n*_ and PI_*n*_ (Fig. 3). These 122 SNPs clustered in 15 different genomic regions that ranged in size from 200 to 561 Kb and were diverse regarding the number of genes (Table 1). Plots for each candidate region, including p-values, genes, local recombination rates, and haplotypic patterns are available in Figure S3 in Supplementary Material. All regions showing high Pygmy-specific differentiation in Mbuti or Biaka were also detected when both Pygmy groups were analyzed together, suggesting the signals were shared (except for region 14 in chromosome 15). Three out of nine regions defined by spatial autocorrelation CI_*n*_ analysis overlapped with the Pygmy PI_*n*_ signals. Interestingly, these three regions (region 5 in chromosome 5, region 9 in chromosome 7 and region 12 in chromosome 10) were among the strongest genomic signals regarding statistical significance and number of significant SNPs.

**Table 1:** Regions covarying with the Pygmy phenotype

Region Id	Chr	Start (Mb)	Region size (Kb)	Genes	Number of significant SNPs in spatial autocorrelation analysis			
					PI_*n*_ Mbuti	PI_*n*_ Biaka	PI_*n*_ Pygmy	CI_*n*_
1	2	31.77	202.4	*MEMO1*			2	
2	2	68.51	228.4	*FBXO48*, ***APLF***, ***PROKR1***		2	3	
3	4	34.42	338.8	–				10
4	4	53.14	302.6	*USP46*, ***SNORA26***, *SCFD2*, *RASL11B*		7	9	
5	5	43.64	467.0	***NNT*** *(FGF10)*	5	7	9	10
6	5	130.84	433.6	***RAPGEF6***, ***FNIP1***, ***ACSL6***				5
7	6	45.18	200.0	***SUPT3H*** **-** ***RUNX2***				1
8	7	118.73	200.0	–				1
9	7	151.43	359.4	*GALNT11,* ***MLL3***, *FABP5L3*, *CCT8L1*		9	8	2
10	8	99.67	230.6	***STK3***				2
11	10	64.57	200.0	*NRBF2*, ***JMJD1C***			1	
12	10	74.44	560.6	*P4HA1*, ***NUDT13***, ***ECD***, ***FAM149B1***, ***DNAJC9***, ***MRPS16***, ***TTC18***, ***ANXA7***, *ZMYND17*, ***PPP3CB***, *USP54*	8		6	11
13	12	8.65	200.0	*AICDA*, *MFAP5*, ***RIMKLB***	1		1	
14	15	62.40	200.0	*CSNK1G1*, *KIAA0101*, ***TRIP4***, *ZNF609*	1			
15	16	49.06	200.0	***NKD1***, *SNX20*				1
				Total	**15**	**25**	**39**	**43**

The regions were defined by including extra 100 Kb in both sides of the significant markers (starting Mb indicated, Build 36.1). The genes within the regions are shown (genes containing significant SNPs are shown in bold and genes nearby are shown in brackets). For more information on the markers defining the regions see Table S3 in Supplementary Material

We checked if the overlap between the two analyses (based on CI_*n*_ and PI_*n*_) observed in the candidate regions was a genomic feature (which would indicate that both statistics are capturing the same signals) or if this overlap was only characteristic of the regions showing extreme height covariation (expected if the phenotype were moderately polygenic but highly differentiated). The genomic correlation between the values of spatial autocorrelation of the two statistics was low (Spearman’s *ρ* = 0.10, *p* < 10^−16^), and increased little at the top 99.9% quantile of the distributions (Spearman’s *ρ* = 0.239, *p* < 10^−7^). This weak correlation showed that the signals captured by the two statistics were independent at a genome-wide level and that genetic differentiation was not sufficient to reach significance in the height covariation analysis.

**Fig. 4:**
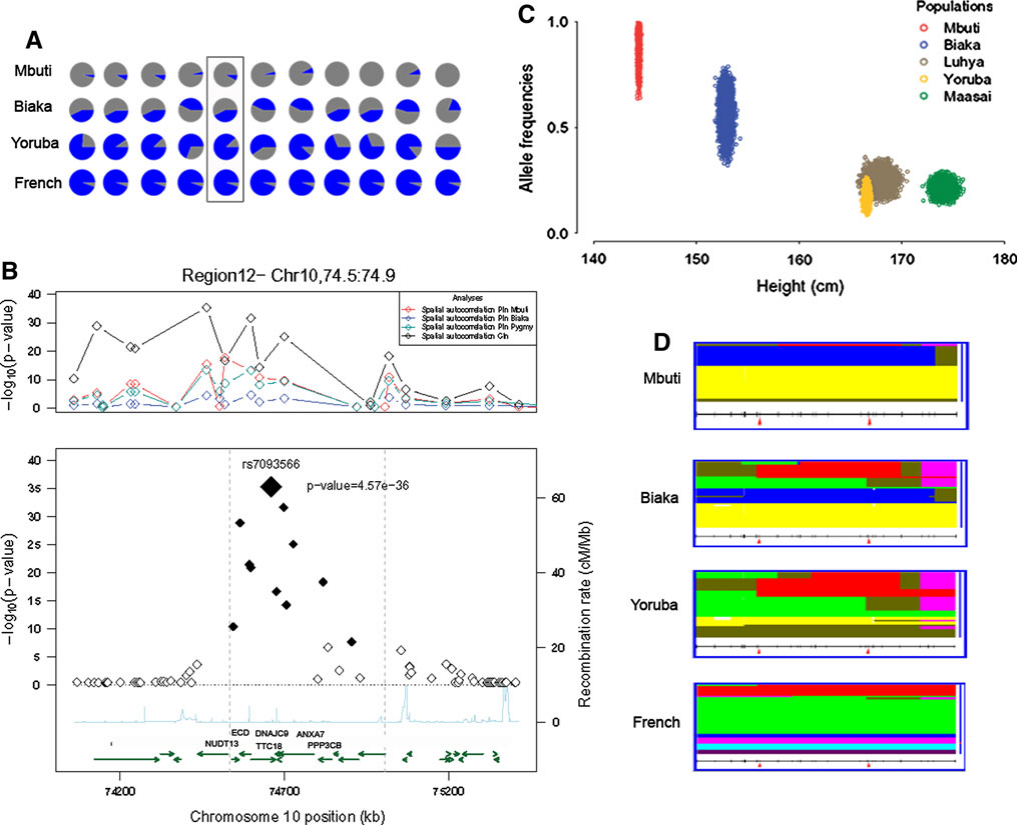
Results found for region 12. **a** Allele frequencies in Pygmies, Yoruba and French populations for the eleven SNPs that were significant in region 12 (rs7093566 within a square). **b** The upper plot shows the −log_10_ (*p* values) for the different analyses at each SNP (represented by *diamonds*) in the area limited by *dashed gray lines* in the lower plot. The lower plot shows the −log_10_ (*p* values) for spatial autocorrelation in CI_*n*_, with the recombination rates and genes in region 12 (the names of the genes not showing significant SNPs were omitted for the sake of clarity, original plot in Figure S3 in Supplementary Material). *Full diamonds* are significant SNPs after Bonferroni correction and the *big diamond* corresponds to the SNP showing the smallest *p* value (indicated). **c** 5,000 samplings from allele frequencies for rs7093566-C were plotted versus height distributions in the five African populations considered in the CI_*n*_ analysis. **d** Haplotype plots for region 12 (marked with *red arrows*) in Mbuti, Biaka, Yoruba and French from HGDP Selection Browser (Coop et al. 2009; Pickrell et al. 2009; Pritchard et al. 2010). Each *row* in the plot is a haplotype, and each *column* is a SNP (*rows* are colored so that all haplotypes of the same color are identical). Plots for all regions are shown in Figure S3 in Supplementary Material (color figure online)

Among the top candidates, the signal in region 5 was located between *NNT* and *FGF10*, genes involved in energy metabolism, and cell growth and survival, respectively. Region 12 showed the strongest signal for height covariation and also strong differentiation in the Mbuti, but not in Biaka Pygmies (see Fig. 4a–c). This discrepancy could be explained by the presence of “non-Pygmy” haplotypes (Fig. 4d), which could mask the differentiation signal in Biaka (in agreement with higher gene flow with non-Pygmy populations and higher average stature). This region includes the *PPP3CB* gene, which encodes for the regulatory beta subunit of calcineurin, a protein that regulates skeletal remodeling through the regulation of osteoblast differentiation. Interestingly, calcineurin inhibitors used as immunosuppressant drugs (such as cyclosporine A) cause acute, rapid and severe bone loss, resulting in up to 65% incidence of fractures (Epstein et al. 1995, 2003; Sun et al. 2005). Finally, regions 9 and 7 contain genes (*MLL3* and *SUPT3H*-*RUNX2*, respectively) that are involved in histone modification, a functional category significantly enriched among height associated genes in genome-wide association studies (GWAS) (Lango Allen et al. 2010). In particular, *SUPT3H*-*RUNX2* genes have repeatedly been associated in both European and Asian populations (Gudbjartsson et al. 2008; Kim et al. 2010; Lango Allen et al. 2010). *RUNX2* is a key transcription factor in osteoblast differentiation, essential for mammalian bone development. Mutations in this gene have been associated with bone development disorder cleidocranial dysplasia (CCD) [MIM 119600]. Intriguingly, overexpression of calcineurin in osteoblasts (gene product of *PPPT3B* gene from region 12 above) increases dramatically the expression levels of RUNX2 protein and enhances bone formation (Sun et al. 2005). The finding of two genes from independent candidate regions interacting in a suggestive pathway for height such as bone remodeling, lends support for their causality.

### Neutral expectation on height covariation and genetic differentiation

Two of the regions detected in this study (region 14 and region 6) were previously reported to have evolved under positive selection (based on extended haplotype homozygosity tests) in African Pygmies (López Herráez et al. 2009). This significant overlap (2 regions versus 0.003 regions expected, *p* = 0.001) suggests that our regions could be enriched for genes under positive selection. In this study, the authors suggested that *TRIP4* (in region 14 in our study) gathers strong functional evidence for a role in the Pygmy phenotype (see discussion in López Herráez et al. 2009).

**Fig. 5:**
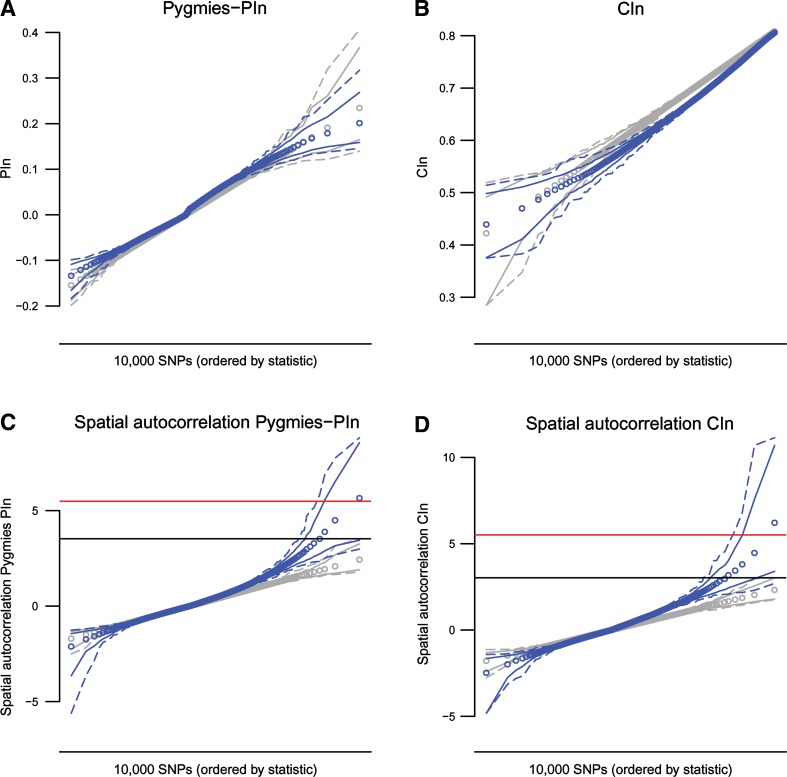
Plots of the statistics for the observed and simulated datasets. Upper plots (**a**, **b**) show the observed (*blue*) and simulated (*gray*) datasets for Pygmies PI_*n*_ and CI_*n*_ statistics, whereas lower plots (**c,** **d**) correspond to the spatial autocorrelation values in the same statistics. *Points* represent mean values, *solid lines* represent the 2.5 and 97.5% quantiles, and the *dashed lines* delimit the maximum and minimum values. In the case of the simulated dataset, the distribution consists on 10,000 SNPs drawn from 1,000 demographic scenarios, and the observed dataset consists on 1,000 samplings of 10,000 SNPs from Illumina dataset used in this study. *Red lines* delimit the significance threshold applied in the study (after Bonferroni correction) and *black lines* point out the maximum value observed in the simulations for the statistics. The results for the PI_*n*_ computed on the Pygmy populations separated (Mbuti and Biaka PI_*n*_) are shown in Figure S4 in Supplementary Material (color figure online)

To confirm the role of selective pressures in the evolution of the ascertained regions, we performed simulations and obtained null distributions of the statistics under plausible demographic scenarios. We observed a high overlap between the simulated and real PI_*n*_ and CI_*n*_ distributions (Fig. 5). In contrast, the spatial autocorrelation values for the same statistics observed in the data consistently presented more extreme values than the simulated ones. The difference was substantially higher for positive spatial autocorrelation values. These results suggest that the most plausible demographic scenarios are compatible with individual markers showing high differentiation or height covariation in Pygmies (according to the overlap of simulated and real PI_*n*_ and CI_*n*_ distributions). However, neither neutral evolution nor purifying selection can explain the presence of clusters of highly differentiated or height covarying SNPs in the genome (spatial autocorrelation distributions). Therefore, the genomic regions located in the upper extreme of the spatial autocorrelation distributions of both PI_*n*_ and CI_*n*_ are strong candidates to have evolved under positive selection in Pygmies.

### Enrichment of genic and non-synonymous variants

As a third independent evaluation of the putative roles of neutral evolution, positive selection and negative selection in the extremes of the statistics, we tested the proportion of genic and non-synonymous (NS) SNPs versus the nongenic ones in the tails of the distributions as in Hancock et al. (2010). Nongenic SNPs are expected to represent neutrally evolving markers whereas genic and NS markers are supposed to have evolved under selective constraints. At three different *p* value cutoffs considered (0.05, 0.1 and 0.15, Table 2), the proportions of genic and NS SNPs versus the nongenic ones were larger than 1 and gradually increasing at more stringent *p* value cutoffs. The genic and NS enrichment was especially high in the distribution of the spatial autocorrelation values of CI_*n*_. Interestingly, the non-synonymous versus nongenic ratios were higher than the genic versus nongenic ones in all significant cases. Although purifying selection could theoretically explain an excess of genic variants, the greater enrichment detected for non-synonymous SNPs compared to genic ones discards the role of purifying selection and fits the expectation under an adaptive scenario (as reviewed in Novembre and Di Rienzo 2009).

**Table 2:** Functional enrichment at the regions covarying with the Pygmy phenotype

Spatial autocorrelation statistics	*p* value cutoff					
	Genic:nongenic			NS:nongenic		
	0.05	0.1	0.15	0.05	0.1	0.15
PI_*n*_ Mbuti	1.66***	1.34*	1.22*	2.34***	1.90***	1.72***
PI_*n*_ Biaka	1.71***	1.23	1.16	1.80*	1.31	1.09
PI_*n*_ Pygmy	1.81***	1.48***	1.36***	1.88**	1.89***	1.57***
CI_*n*_	2.13***	1.74***	1.64***	2.53***	2.19***	1.91***

Ratios of the genic and non-synonymous (NS) SNPs relative to the nongenic SNPs in different tails of the distributions of the statistics defined by the upper *p* value cutoffs indicated (0.05, 0.1 and 0.15)

* Bootstrapping significance at 5% level

** Bootstrapping significance at 2.5% level

*** Bootstrapping significance at 1% level

### Enrichment of functional pathways

To unravel the biological pathways responsible for the putative adaptive evolution of the candidate regions, we subjected the 33 genes strongly differentiated in Pygmies (PI_*n*_) and the 24 genes that strongly covaried with height (CI_*n*_) (genes listed in Table 1) to the ingenuity pathway analysis (IPA). We found that the highly differentiated gene set was significantly enriched for three pathways: two of them involved in the general cellular processes “protein ubiquitination” and “*O*-glycan biosynthesis”, and the immune related pathway “role of NFAT in regulation of the immune response” (Figure S5 in Supplementary Material). Conversely, in the case of the height covarying genes the strongest pathway identified was “role of osteoblasts, osteoclasts and chondrocytes in rheumatoid arthritis” (Fisher’s exact test, *p* = 3.37 × 10^−2^), whereas the other two significant signals were shared with the highly differentiated gene set (“*O*-glycan biosynthesis” and “protein ubiquitination”). The finding of significant enrichment of genes involved in bone remodeling in the regions that strongly covary with the phenotypic trait constitutes a strong hint for a putative role of this pathway in the African Pygmy phenotype.

## Discussion

Pygmies are a group of genetically heterogeneous human populations characterized by extreme short statures in comparison to other humans (Cavalli-Sforza 1986). Despite the visibility of the trait and the genetic evidence of its inheritance, little is known about the genetic architecture of this phenotype (Perry and Dominy 2009). Although adaptation seems the most parsimonious explanation for the presence of the Pygmy phenotype in different continents, the adaptive value of the trait has not been confirmed. In the present approach, we studied the genomic covariation with height in Pygmy and non-Pygmy African populations. Under the assumption that the genetic basis of the phenotype covaries with the trait and is shared among populations, we explored the association between genetic and phenotypic data at a population level.

We observed that in the case where the genetic architecture of Pygmy height was similar to that of normal height variation, with thousands of causal markers (Yang et al. 2010), this population-based study would not be sufficiently powered. Conversely, if the phenotype were polygenic, but the causal variants were in number of hundreds and showed strong population differentiation, the extremes of the empirical distribution of our conditional informativeness (CI_*n*_) statistic would be enriched for true causal variants. Nevertheless, the statistic has several limitations. First, it assumes that the genetic basis of the phenotype is, at least partially, shared by all the populations studied and neglects the effect of genetic interactions. Second, spurious signals in CI_*n*_ analysis could occur under (1) the presence of genetic variants associated with other phenotypes with a geographic distribution similar to height in these populations or (2) allele frequency clines mimicking the distribution of the phenotype due to genetic drift. Nonetheless, the inclusion of several African populations with very different demographic histories (counteracting drift effects), and the study of spatial autocorrelation with extremely conservative significance cutoffs could be expected to reduce these spurious signals.

Because the assumption of shared genetic basis among African populations could be violated in the case of the Pygmy phenotype, we pursued a second analysis with no premises regarding the phenotypic model. We computed the *Pygmy*-*specific informativeness* (PI_*n*_) to identify markers that are highly differentiated in African Pygmies when compared with other worldwide populations. In addition, we studied the genomic distribution of the two statistics by means of spatial autocorrelation to identify clusters of markers showing strong differentiation and/or association with the phenotype.

Some of the ascertained regions harbored genes that were previously described as being under positive selection in African Pygmies, suggesting these loci could be enriched in adaptive genes. To confirm whether our statistics would be empowered to detect non-neutral evolving markers, we performed simulations to learn about the distribution expected under the most plausible demographic scenarios. Even though demography can be a confounding factor when assessing the adaptive value of highly differentiated or height covarying markers, we showed that it is very unlikely that demographic processes alone could create haplotypes like the ones observed at the extremes of the spatial autocorrelation distributions. Because neither the LD pattern nor the genome-wide distribution of PI_*n*_ and CI_*n*_ from simulations differed from real SNP data, and since purifying selection cannot explain these patterns, these suggests an enrichment of loci that have undergone positive selection. In addition, the substantial functional excess found in the extreme of the distributions of the statistics (particularly of non-synonymous SNPs) also points at positive selection as the most likely force for explaining this disproportion.

The selective scenario fits well within the finding that the regions that most strongly covaried with height distributions showed extreme differentiation. This suggests that the genetic architecture of the phenotype could be constituted by a limited set of adaptive genes with moderate to large phenotypic effect. The list of candidate regions includes well-known height associated loci in other human populations and an excess of genes involved in skeletal remodeling. The functional pathway enrichment analysis supported our approach, as the incorporation of population–phenotypic information allowed to distinguish regions more likely to be associated with the trait of interest from adaptive signals corresponding to other traits (such as immunity).

The almost complete sharing of signals between the two Pygmy groups suggests that the phenotype is not the consequence of convergent evolution and so is older than the split between Eastern and Western Pygmies (25,000 years). We found no evidence for a role of genes within the *GH*-*IGF* pathway, which could be due to an incomplete coverage in those genes or that genetic variants in the *GH*-*IGF* pathway are not relevant in the phenotype. Not being able to completely discard the role of the growth hormone pathway, our results suggest a predominant role of bone remodeling in the etiology of the Pygmy phenotype. Interestingly, recent research has shown functional links between bone remodeling, energy metabolism and fertility (Oury et al. 2011; Schuh-Huerta and Pera 2011), which does not allow us to prioritize among the different (and non-mutually exclusive) hypotheses regarding the adaptive value of the Pygmy size. Alternatively, this could constitute a piece of evidence in favor of combined effects of both habitat-specific ecology and life history on the adaptive benefits of the Pygmy phenotype. Further functional characterization of the reported regions is imperative to assess the phenotypic contribution of each of the candidate regions we propose in this population-based study.

## Conclusions

During the last few years, most scans for human adaptations have focused on genetic data alone, ignoring the information that population-based phenotypes may provide. Instead, our approach goes along the lines of recent efforts that have focused in incorporating environmental variables in the genetic analysis to understand local adaptation (Coop et al. 2009; Hancock et al. 2008, 2010). We open up the possibility to study the genetics of differentiated phenotypes (not necessarily adaptive) when the phenotypic and genotypic variation are only known at a population level. This approach appears appealing in the current context in which public genetic data from many worldwide populations is available but there is no phenotypic information for the genotyped individuals. By applying our novel approach we have been able to show that the Pygmy phenotype is most likely moderately polygenic and adaptive. Furthermore, we have been able to gain insights into the genomic regions that most likely participate in the genetic architecture of the phenotype, pointing at bone homeostasis as an underlying biological function. These results constitute the first genetic hint of adaptive evolution in the African Pygmy phenotype, consistent with independent emergences of the Pygmy stature in human evolution.

## Electronic supplementary material

Below is the link to the electronic supplementary material.

Supplementary material 1 (DOC 3035 kb)
