# Supplementary material for: Adaptive evolution of loci covarying with the human African Pygmy phenotype
Source: Hum Genet. 2012 Mar 11;131(8):1305–17. doi: 10.1007/s00439-012-1157-3 (PMC3397127; doi:10.1007/s00439-012-1157-3)
Supplement: Supplementary file 1 — Supplementary material 1 (DOC 3035 kb) [file 439_2012_1157_MOESM1_ESM.doc]

**Adaptive evolution of loci covarying with the human African Pygmy Phenotype**

*Human Genetics (Original investigation)*

Isabel Mendizabal^1^, Urko M. Marigorta^1^, Oscar Lao^2,3^ and David Comas^1,3^

^1^Institut de Biologia Evolutiva (CSIC-UPF), Departament de Ciències de la Salut i de la Vida, Universitat Pompeu Fabra, 08003 Barcelona, Spain.

^2^Department of Forensic Molecular Biology, Erasmus MC University Medical Center Rotterdam, 3015 GE Rotterdam, The Netherlands.

^3^ These authors contributed equally to this work

**Corresponding author:**

Oscar Lao

Department of Forensic Molecular Biology

Erasmus MC University Medical Center Rotterdam

3015 GE Rotterdam, The Netherlands

Telephone number: 0031107042996

Fax number: 0031107044575

e-mail address: [o.laogrueso@erasmusmc.nl](mailto:o.laogrueso@erasmusmc.nl)

**Electronic Supplementary Material 1**

[**Supplemental Methods**](#suppmaterialup)**.** Pages 2-4

Conditional Informativeness statistic. Page 2

Demographic simulations**.** Pages 3-4

**Supplemental Tables.** Pages 5-12

[Table S1](#tableS1). Genotype and phenotype data for the analysis of Conditional Informativeness. Page 5

[Table S2.](#tableS3) Genetic architectures tested for the Pygmy phenotype. Page 6

[Table S3.](#tablahiru) Significant markers and regions. Pages 7-9

[Table S4.](#tablalau) Prior distributions, posterior distributions and accuracy estimates of the ABC. Page 10

[Table S5](#tableS5). List of 400 STRs used for the ABC analysis. Pages 11-12

**Supplemental Figures.** Pages 13-28

[Figure S1.](#figureS1) Validation of the Conditional Informativeness statistic. Pages 13-14

[Figure S2.](#FigureS5) Demographic simulations. Page 15

[Figure S3.](#s2) Genes, recombination rates, p-values and haplotype plots for the candidate regions. Pages 16-27

[Figure S4.](#realfigures4) Plots of the statistics for the observed and simulated datasets. Page 28

[Figure S5**.**](#references) Results for the Ingenuity Pathway Analysis. Page 29

**Supplemental References.** Pages 30-31

[**Supplemental Methods**](#_top)

**Conditional Informativeness statistic**

Because I_n_ is a derived statistic from the concept of mutual information (Cover and Thomas 1991), we used the chain rule for mutual information statistic (see Theorem 2.5.2 in Cover and Thomas (1991)) to define how much extra information the phenotype (*P*) contains to infer the geographic origin of one individual after removing the information that is provided by knowing its genotype (*J*):

$$\text{CI}_{\text{n}}\text{=I}_{\text{n}}\text{(Q;P|J)=I}_{\text{n}}\text{(Q;P;J)-I}_{\text{n}}\text{(Q;J)}$$

A limitation of this statistic is that the actual height values and genotypes for the same individuals (joint distribution of the phenotype and genotype (*P*(*J*;*P*)) are unknown. Assuming individual independence of *J* and *P* within each population, we can compute I_n_(*Q*;*P*;*J*) by multiplying category frequencies over all possible phenotype-genotype combinations within each population (Cover and Thomas 1991; Rosenberg et al. 2003). However, since the phenotype is continuous, integration is required. Here, we applied a Monte Carlo algorithm based on the Asymptotic Equipartition Property of Entropy, AEP (Cover and Thomas 1991), to compute I_n_(*Q*;*P*;*J*). Specifically, for a given SNP and for each population, we obtained a combination of a genotype (assuming HWE and the allele frequencies based on real genotype data) and a height value (by sampling from the normal distribution with the mean height and SD of the population). For each sampled combination in one population we obtained its I_n_ (*Q*;*P*;*J*) by estimating the allelic and phenotypic frequencies in all populations. Repeating the process 5,000 times per population, we obtained the distribution of I_n_(*Q*;*P*;*J*) values and, by AEP, we assumed that the average over all the sampled I_n_(*Q*;*P*;*J*) is an unbiased estimation of the true value of I_n_(*Q*;*P*;*J*). In total, 25,000 phenotype-genotype samples per SNP were obtained.

**Demographic simulations**

The data used for the ABC analysis (see models tested in Figure S3) consisted on 400 autosomal short tandem repeats (STRs) with 4-bp motifs genotyped on the CEPH (Rosenberg 2006; Rosenberg et al. 2005) with the minimum sample size common to all populations (13 Mbuti, Biaka and Yoruba individuals). We did not include the Eastern populations (Luhya and Maasai) in the ABC due to their more complicated demographies (Campbell and Tishkoff 2010). The choice of STR markers instead of SNPs was motivated to avoid the effect of ascertainment bias in SNP data (Clark et al. 2005). We excluded STRs that have been reported as candidates for local selection (Kayser et al. 2003; Storz et al. 2004), and selected those with less than 3% of missing data and lowest percentage of imperfect repeats (Table S5). The summary statistic used in the was the modified Hausdorff distance (Dubuisson and Jain 1994) computed between the simulated and observed Multidimensional Scaling (MDS) plots (de Gruijter et al. 2011), since it has been shown that principal components analysis (PCA) can be interpreted in evolutionary terms (McVean 2009), and PCA and classical MDS plots based on identity-by-state (IBS) matrices are equivalent (Wang et al. 2010). For each STR, we normalized the observed repeat length distribution by using the inverse of a normal distribution and used these standardized measures to compute absolute distances between pairs of individuals. This distance matrix was then used to perform a MDS analysis considering two dimensions. Because it is the relative distribution of the individuals which defines the relationship, a previous rotation of the first and second dimensions was required in order to make two different MDS plots comparable. After the transposition, we computed a modified Hausdorff distance for the cloud of points of each population between the observed and the simulated. This distance took value 0 only when two clouds of points perfectly overlapped. Prior distributions of the parameters consisted on wide flat distributions (Table S3). We used Simcoal 2.0 software (Laval and Excoffier 2004) to generate 500,000 simulations randomly drawing the model parameters from prior distributions. Applying the rejection algorithm (2002), we retained 1,000 simulations (tolerance 0.002). The dataset of 400 autosomal STRs was not informative enough to differentiate between the best two models tested by ABC. Therefore, we assumed that ((Mbuti,Biaka),Yoruba) was the correct model (as reported by previous studies as the most probable one (Batini et al. 2011; Patin et al. 2009)) and repeated the ABC by fixing the splitting time of Yoruba and scaling the rest of the parameters according to it as log(parameter/time split Yoruba). The time considered for the split of the Yoruba from the ancestor of Pygmies was 60Ky (Batini et al. 2011; Patin et al. 2009; Verdu et al. 2009; Wegmann et al. 2009).

Subsequently, in order to get the null demographic distributions of the statistics, we drew 1,000 values from the posterior distributions of each parameter (Table S3). This set of most probable demographic parameters was used to generate 1,000 genomic stretches of 5Mb each (5 Gb in total) with Cosi simulator (default mutation rate and genetic recombination map (Schaffner et al. 2005)). Since our ABC analysis did not include European populations, we included Schaffner’s estimation of the European demographic parameters (Schaffner et al. 2005). From each simulated stretch of 5Mb, 1,000 SNPs were recovered for further analyses. These SNPs were chosen to fulfil different aspects of the Illumina dataset including (i) SNP density of 1 per 5Kb (ii) SNP spacing (iii) MAF spectrum and (iv) LD block length, which could be crucial for allowing comparison of the Local Moran’s I statistic (see Figure S3). Finally, we computed the statistics PI_n_ and CI_n_ and their spatial autocorrelation values in the ascertained SNPs, performed 1,000 random samplings of 10,000 SNPs and obtained the significance thresholds of differentiation that the most probable demographic scenarios support. Since the demographic parameters for the Maasai and Luhya were not simulated, the CI_n_ statistic was computed on Mbuti, Biaka and Yoruba, both in the simulated and the real data used for comparison.

**Supplemental Tables**

[**Table S1**](#_top)**. Data used in the Conditional Informativeness is shown in the table.**

On the left side of the table information on the genotypic is given whereas the corresponding height measurements for adult males from the same populations are shown in the right. D.R.C: Democratic Republic of Congo; C.A.R.: Central African Republic.

| **Genotypic Data** | | | | **Phenotypic Data** | | | | | |
| --- | --- | --- | --- | --- | --- | --- | --- | --- | --- |
| **Population** | **Origin** | **N** | **Ref.** | **Population** | **Origin** | **N** | **Adult male**  **height (cm)** | | **Ref.** |
|  |  |  |  |  |  |  | Mean | S.D. |  |
| Mbuti | Ituri, D.R.C | 13 | (Li et al. 2008) | Mbuti | Ituri, D.R.C. | 71 | 144.4 | 0.54 | (Cavalli-Sforza 1986) |
| Biaka | C.A.R. | 21 | (Li et al. 2008) | Biaka | Bagandu, C.A.R. | 427 | 152.9 | 6.4 | (Cavalli-Sforza 1986) |
| Luhya | Kenya | 82 | (Altshuler et al. 2010) | Kamba | Kenya | 106 | 167.9 | 6.7 | (Christensen et al. 2008) |
| Yoruba | Nigeria | 107 | (Altshuler et al. 2010) | Yoruba | Nigeria | 2455 | 166.6 | 6.5 | (Eltis 1982) |
| Maasai | Kenya | 139 | (Altshuler et al. 2010) | Maasai | Kenya | 172 | 174.1 | 6.7 | (Christensen et al. 2008) |

[**Table S**](#_top)**2**. G**enetic architectures tested for the Pygmy phenotype.**

The rows indicate the number of causal markers considered and the columns indicate the phenotypic effect of each allele copy (cm) under an additive model. The number within each cell indicates the mean height differences between simulated Maasai and Mbuti individuals according to the genetic architecture considered (given a number of SNPs and a phenotypic effect). For the first scenario, the SNPs were taken randomly from the genome and considered only if the allele frequencies followed the phenotypic cline (Mbuti ≤ Biaka < Yoruba/Luhya < Maasai). For the second scenario the causal SNPs were forced to also show allele frequency differences between Maasai and Mbuti (delta) greater than 0.37, corresponding to the 95% quantile. The architectures showing mean differences between Maasai and Mbuti close to the real value (30cm) where chosen to run the CI_n_ statistic.

|  | **Phenotypic effect (cm)** | | | | | | | | | | | | | | | |
| --- | --- | --- | --- | --- | --- | --- | --- | --- | --- | --- | --- | --- | --- | --- | --- | --- |
| **Number of SNPs** | **0.01** | **0.02** | **0.05** | **0.1** | **0.2** | **0.5** | **1** | **2** | **3** | **4** | **5** | **6** | **7** | **8** | **9** | **10** |
| ***Scenario 1: allele frequencies follow the phenotypic pattern*** | | | | | | | | | | | | | | | | |
| **3** | 0.02 | 0.04 | 0.09 | 0.18 | 0.35 | 0.90 | 1.77 | 3.46 | 5.41 | 7.12 | 8.92 | 10.12 | 11.80 | 14.45 | 15.37 | 17.57 |
| **5** | 0.02 | 0.05 | 0.13 | 0.23 | 0.48 | 1.23 | 2.51 | 5.04 | 7.63 | 10.17 | 12.67 | 15.32 | 17.42 | 20.12 | 21.58 | 25.79 |
| **10** | 0.06 | 0.11 | 0.28 | 0.56 | 1.13 | 2.77 | 5.54 | 11.18 | 16.57 | 23.02 | 28.01 | 34.01 | 39.57 | 44.52 | 51.63 | 58.02 |
| **15** | 0.08 | 0.17 | 0.42 | 0.84 | 1.70 | 4.13 | 8.42 | 17.28 | 25.21 | 33.60 | 42.05 | 50.77 | 58.60 | 67.53 | 77.16 | 83.50 |
| **20** | 0.11 | 0.23 | 0.56 | 1.13 | 2.25 | 5.63 | 11.37 | 22.84 | 34.09 | 44.81 | 57.22 | 68.09 | 78.88 | 89.74 | 100.50 | 111.16 |
| **25** | 0.14 | 0.29 | 0.73 | 1.45 | 2.90 | 7.41 | 14.94 | 29.90 | 43.56 | 58.61 | 73.25 | 88.42 | 101.79 | 116.31 | 129.81 | 147.44 |
| **50** | 0.28 | 0.55 | 1.38 | 2.78 | 5.54 | 13.75 | 27.76 | 55.34 | 82.81 | 111.03 | 138.24 | 164.53 | 196.95 | 219.94 | 250.79 | 275.84 |
| **100** | 0.57 | 1.13 | 2.83 | 5.64 | 11.38 | 28.22 | 56.99 | 113.58 | 170.60 | 226.11 | 285.26 | 339.87 | 395.23 | 454.74 | 509.99 | 566.10 |
| **200** | 1.14 | 2.30 | 5.76 | 11.42 | 22.90 | 57.28 | 114.47 | 228.20 | 341.84 | 457.58 | 572.71 | 685.25 | 801.23 | 915.67 | 1033.98 | 1141.44 |
| **300** | 1.67 | 3.35 | 8.37 | 16.72 | 33.30 | 83.84 | 167.22 | 335.21 | 502.90 | 669.82 | 833.14 | 1003.58 | 1171.06 | 1340.03 | 1501.30 | 1672.28 |
| **500** | 2.80 | 5.62 | 14.04 | 28.10 | 56.01 | 140.44 | 281.12 | 562.30 | 844.21 | 1126.22 | 1406.73 | 1678.25 | 1966.66 | 2244.74 | 2535.99 | 2809.80 |
| **1000** | 5.64 | 11.24 | 28.16 | 56.34 | 112.44 | 281.60 | 562.77 | 1127.09 | 1693.19 | 2252.68 | 2818.88 | 3368.74 | 3941.01 | 4498.75 | 5055.06 | 5636.32 |
| ***Scenario 2: allele frequencies follow the phenotypic pattern and delta Maasai-Mbuti >0.37*** | | | | | | | | | | | | | | | | |
| **3** | 0.03 | 0.05 | 0.14 | 0.28 | 0.54 | 1.39 | 2.78 | 5.44 | 8.19 | 11.02 | 13.63 | 16.67 | 18.96 | 21.42 | 24.37 | 28.05 |
| **5** | 0.05 | 0.09 | 0.23 | 0.45 | 0.91 | 2.30 | 4.47 | 8.87 | 13.55 | 18.56 | 22.58 | 27.83 | 32.39 | 36.37 | 41.17 | 45.26 |
| **10** | 0.09 | 0.18 | 0.45 | 0.92 | 1.85 | 4.64 | 9.24 | 18.23 | 27.70 | 36.62 | 45.23 | 55.27 | 63.13 | 72.82 | 82.75 | 91.74 |
| **15** | 0.14 | 0.27 | 0.69 | 1.36 | 2.76 | 6.81 | 13.76 | 27.52 | 40.85 | 54.81 | 68.62 | 83.57 | 97.50 | 109.69 | 123.10 | 137.07 |
| **20** | 0.18 | 0.36 | 0.91 | 1.82 | 3.64 | 9.07 | 18.20 | 36.77 | 54.42 | 73.44 | 92.37 | 109.40 | 127.32 | 146.86 | 162.49 | 182.43 |
| **25** | 0.23 | 0.46 | 1.15 | 2.29 | 4.58 | 11.35 | 22.58 | 45.36 | 68.64 | 91.25 | 113.21 | 137.41 | 161.46 | 182.10 | 205.81 | 227.83 |
| **50** | 0.45 | 0.91 | 2.27 | 4.55 | 9.04 | 22.82 | 45.68 | 90.99 | 136.29 | 180.06 | 228.05 | 272.45 | 317.77 | 365.00 | 411.29 | 456.43 |
| **100** | 0.92 | 1.83 | 4.57 | 9.10 | 18.16 | 45.49 | 91.31 | 182.02 | 272.96 | 361.15 | 455.88 | 545.11 | 640.19 | 730.60 | 817.54 | 910.03 |
| **200** | 1.81 | 3.61 | 9.04 | 18.10 | 36.34 | 90.54 | 180.46 | 363.27 | 544.37 | 724.83 | 906.00 | 1086.78 | 1267.83 | 1445.22 | 1629.68 | 1814.31 |
| **300** | 2.72 | 5.44 | 13.58 | 27.24 | 54.19 | 135.70 | 271.90 | 542.67 | 814.55 | 1086.98 | 1358.38 | 1634.03 | 1904.90 | 2176.72 | 2451.07 | 2732.58 |
| **500** | 4.51 | 9.06 | 22.59 | 45.09 | 90.12 | 226.08 | 452.47 | 904.05 | 1356.74 | 1807.56 | 2256.09 | 2712.55 | 3163.54 | 3610.66 | 4068.84 | 4516.80 |
| **1000** | 9.03 | 18.07 | 45.27 | 90.37 | 180.80 | 452.13 | 903.31 | 1807.06 | 2710.70 | 3618.16 | 4525.30 | 5430.06 | 6333.87 | 7240.42 | 8139.83 | 9035.44 |

[**Table S**](#_top)**3. Significant markers and regions.**

Significant markers and regions are shown with the corresponding p-values, as well as the information of type of SNP regarding the consequence to transcript according to Ensembl Variation 59. PI_n_ stands for the Pygmy-specific Informativeness for assignment, CI_n_ stands for Conditional Informativeness for assignment CI_n_, LMI stands for the spatial autocorrelation (Local Moran’s I statistic). The genes in bold are markers significant for the corresponding analyses after Bonferroni correction (The limit p-value significance are 1.35E-07, 1.16E-07, 1.37E-07, 1.06E-07 for spatial autocorrelation in PI_n_ Biaka, PI_n_ Mbuti, PI_n_ Pygmy and CI_n_ respectively). NA indicates non-available data.

|  |  |  |  |  |  |  |  | **p.value** |  |  |
| --- | --- | --- | --- | --- | --- | --- | --- | --- | --- | --- |
| **Id** | **Chr** | **Start (Mb)** | **Size (Kb)** | **SNPs** | **Gene** | **SNP type (consequence to transcript)** | **LMI-PI_n_**  **Mbuti** | **LMI- PI_n_**  **Biaka** | **LMI-PI_n_ Pygmy** | **LMI-CI_n_** |
| 1 | 2 | 31.9 | 2.4 | rs223655 |  | INTERGENIC | 1.19E-05 | 8.91E-05 | **1.73E-08** | 1.43E-05 |
|  |  |  |  | rs223657 |  | DOWNSTREAM | 3.78E-05 | 2.72E-04 | **1.14E-07** | 3.44E-04 |
| 2 | 2 | 68.6 | 28.4 | rs7423646 | *^1^APLF;^2^PROKR1* | ^1^INTRONIC;^2^UPSTREAM | 1.44E-04 | 4.97E-05 | **6.80E-08** | 7.14E-05 |
|  |  |  |  | rs6724540 | *APLF;PROKR1* | INTRONIC | 2.64E-06 | **6.04E-09** | **6.83E-13** | 1.51E-05 |
|  |  |  |  | rs6546418 | *APLF;PROKR1* | INTRONIC | 1.12E-03 | **2.78E-08** | **7.55E-09** | 4.57E-04 |
| 3 | 4 | 34.5 | 138.8 | rs16990385 |  | INTERGENIC | 4.13E-04 | 3.67E-01 | 4.88E-02 | **4.38E-13** |
|  |  |  |  | rs16990420 |  | INTERGENIC | 3.66E-01 | NA | NA | **1.38E-08** |
|  |  |  |  | rs11730487 |  | INTERGENIC | 1.04E-01 | NA | 3.59E-01 | **4.41E-08** |
|  |  |  |  | rs6831060 |  | INTERGENIC | 2.65E-03 | 3.72E-01 | 1.34E-01 | **1.89E-09** |
|  |  |  |  | rs11944089 |  | INTERGENIC | 5.50E-03 | 3.74E-01 | 1.54E-01 | **1.95E-08** |
|  |  |  |  | rs10517336 |  | INTERGENIC | 3.32E-04 | 3.83E-01 | 1.42E-01 | **5.48E-13** |
|  |  |  |  | rs6854453 |  | INTERGENIC | 1.13E-01 | NA | NA | **8.91E-09** |
|  |  |  |  | rs11735190 |  | INTERGENIC | 5.39E-03 | 3.80E-01 | 1.68E-01 | **2.20E-11** |
|  |  |  |  | rs874936 |  | INTERGENIC | 5.29E-04 | NA | 2.90E-01 | **4.71E-09** |
|  |  |  |  | rs16990598 |  | INTERGENIC | 6.49E-04 | NA | 2.50E-01 | **5.23E-09** |
| 4 | 4 | 53.2 | 102.6 | rs746687 |  | INTERGENIC | 6.11E-03 | **2.27E-08** | **1.29E-08** | 1.87E-06 |
|  |  |  |  | rs12505070 |  | INTERGENIC | 3.56E-04 | **3.09E-11** | **3.93E-12** | NA |
|  |  |  |  | rs10028122 | *SNORA26* | UPSTREAM | 3.94E-06 | **1.06E-11** | **4.11E-14** | 1.71E-07 |
|  |  |  |  | rs7677282 | *SNORA26* | DOWNSTREAM | 7.51E-06 | **1.21E-06** | **2.79E-10** | 2.84E-05 |
|  |  |  |  | rs6836873 |  | WITHIN_NON_CODING_GENE | 1.63E-05 | **8.59E-12** | **9.06E-14** | 5.34E-06 |
|  |  |  |  | rs6840875 |  | WITHIN_NON_CODING_GENE | 1.61E-02 | **1.79E-12** | **2.10E-11** | 3.83E-04 |
|  |  |  |  | rs4865399 |  | WITHIN_NON_CODING_GENE | 1.61E-02 | **1.87E-12** | **2.14E-11** | 1.81E-04 |
|  |  |  |  | rs10009096 |  | INTERGENIC | 3.76E-04 | **2.26E-10** | **2.87E-11** | 1.38E-05 |
|  |  |  |  | rs10000595 |  | INTERGENIC | 1.01E-03 | 1.75E-06 | **1.02E-07** | 4.78E-04 |
| 5 | 5 | 43.7 | 267.0 | rs10069201 | *NNT* | DOWNSTREAM | 2.84E-02 | 2.46E-02 | 1.26E-03 | **5.95E-11** |
|  |  |  |  | rs10062920 |  | 3 PRIME UTR | 1.52E-05 | 5.09E-06 | **1.56E-09** | **1.22E-20** |
|  |  |  |  | rs4449542 |  | INTERGENIC | **5.25E-08** | **3.19E-08** | **3.34E-13** | **1.62E-29** |
|  |  |  |  | rs7721405 |  | INTERGENIC | **1.39E-08** | **8.58E-09** | **3.84E-14** | **3.86E-29** |
|  |  |  |  | rs6875400 |  | INTERGENIC | **1.40E-08** | **3.38E-09** | **1.58E-14** | **2.53E-30** |
|  |  |  |  | rs10045432 |  | INTERGENIC | 5.10E-03 | **7.87E-08** | **5.16E-09** | **2.08E-12** |
|  |  |  |  | rs7729400 |  | WITHIN_NON_CODING_GENE | 1.49E-07 | **2.74E-08** | **4.28E-12** | **2.44E-30** |
|  |  |  |  | rs7735852 |  | INTERGENIC | **1.87E-11** | **2.80E-11** | **5.11E-17** | **2.08E-30** |
|  |  |  |  | rs4866756 |  | INTERGENIC | **8.18E-10** | **2.22E-13** | **5.58E-18** | **6.08E-26** |
|  |  |  |  | rs7721923 |  | INTERGENIC | 1.15E-05 | 1.12E-05 | **1.68E-08** | **1.82E-12** |
| 6 | 5 | 130.9 | 233.6 | rs10477734 | *RAPGEF6; FNIP1* | INTRONIC | 1.63E-01 | 3.98E-01 | 3.49E-01 | **1.33E-15** |
|  |  |  |  | rs7706785 | *RAPGEF6; FNIP1* | INTRONIC | 2.66E-01 | 3.56E-01 | 3.90E-01 | **1.61E-16** |
|  |  |  |  | rs1295873 | *RAPGEF6; FNIP1* | INTRONIC | 3.79E-01 | 3.98E-01 | 3.87E-01 | **1.17E-22** |
|  |  |  |  | rs26008 | *FNIP1* | NON_SYNONYMOUS_CODING | 3.83E-01 | 3.99E-01 | 3.71E-01 | **2.78E-20** |
|  |  |  |  | rs924434 | *ACSL6* | INTRONIC | 3.98E-01 | 2.12E-01 | NA | **8.76E-11** |
| 7 | 6 | 45.3 | - | rs9463076 | *SUPT3H* | INTRONIC | 3.20E-02 | NA | 3.97E-01 | **4.30E-09** |
| 8 | 7 | 118.8 | - | rs7798719 |  | INTERGENIC | 3.59E-01 | NA | NA | **2.20E-09** |
| 9 | 7 | 151.5 | 159.4 | rs10278844 | *MLL3* | INTRONIC | 9.48E-03 | 1.24E-02 | 1.74E-04 | **3.00E-08** |
|  |  |  |  | rs6943984 | *MLL3* | INTRONIC | 9.47E-03 | 1.24E-02 | 1.74E-04 | **4.08E-08** |
|  |  |  |  | rs7796107 |  | INTRONIC | 3.25E-03 | **1.75E-08** | **2.48E-09** | 1.98E-04 |
|  |  |  |  | rs10248857 | *MLL3* | INTRONIC | 1.97E-02 | **7.98E-11** | **1.24E-09** | 2.30E-04 |
|  |  |  |  | rs7786732 | *MLL3* | INTRONIC | 4.12E-02 | **1.04E-07** | 3.16E-07 | 2.84E-03 |
|  |  |  |  | rs10239783 | *MLL3* | INTRONIC | 5.22E-02 | **6.48E-09** | **7.95E-08** | 3.80E-03 |
|  |  |  |  | rs10249694 | *MLL3* | INTRONIC | 1.57E-02 | **6.42E-09** | **1.23E-08** | 2.01E-04 |
|  |  |  |  | rs7799662 | *MLL3* | INTRONIC | 1.13E-02 | **2.07E-08** | **1.61E-08** | 7.11E-04 |
|  |  |  |  | rs10279901 | *MLL3* | INTRONIC | 4.12E-02 | **2.08E-08** | **1.16E-07** | 1.58E-03 |
|  |  |  |  | rs10263545 | *MLL3* | INTRONIC | 3.80E-02 | **1.56E-08** | **7.87E-08** | NA |
|  |  |  |  | rs10226650 | *MLL3* | INTRONIC | 1.13E-02 | **2.09E-08** | **1.62E-08** | NA |
| 10 | 8 | 99.8 | 30.6 | rs7818828 | *STK3* | INTRONIC | 5.72E-03 | 3.89E-01 | 3.64E-01 | **5.53E-12** |
|  |  |  |  | rs6988087 | *STK3* | INTRONIC | 3.50E-02 | 3.87E-01 | 3.78E-01 | **9.60E-09** |
| 11 | 10 | 64.7 | - | rs6479890 | *JMJD1C* | INTRONIC | 6.27E-04 | 2.64E-07 | **3.93E-09** | 4.13E-04 |
| 12 | 10 | 74.5 | 360.6 | rs4492736 | *NUDT13* | INTRONIC | 1.05E-03 | 1.11E-01 | 2.91E-03 | **4.24E-11** |
|  |  |  |  | rs2271904 | *NUDT13^1^; ECD^2^* | ^1^DOWNSTREAM;^2^NON_SYNONYMOUS_CODING | 4.39E-0 | 2.94E-02 | 2.91E-05 | **1.37E-29** |
|  |  |  |  | rs3812619 | *ECD; FAM149B1* | ^1^NON_SYNONYMOUS_CODING;^2^UPSTREAM | **3.25E-09** | 3.49E-02 | 1.70E-06 | **3.27E-22** |
|  |  |  |  | rs3763723 | *ECD; FAM149B1* | ^1^INTRONIC;^2^UPSTREAM | **3.39E-09** | 3.49E-02 | 1.71E-06 | **1.20E-21** |
|  |  |  |  | rs7093566 | *FAM149B1; DNAJC9* | ^1^INTRONIC;^2^WITHIN_NON_CODING_GENE | **3.79E-16** | 3.61E-05 | **3.76E-14** | **4.57E-36** |
|  |  |  |  | rs12258130 | *FAM149B1^1^; DNAJC9^2^;MRPS16^3^;* | ^1^DOWNSTREAM;^2^WITHIN_NON_CODING_GENE;^3^DOWNSTREAM | **1.70E-18** | 4.99E-02 | **2.41E-09** | **2.44E-17** |
|  |  |  |  | rs11000579 | *TTC18* | INTRONIC | **6.21E-14** | 2.48E-05 | **5.91E-14** | **2.40E-32** |
|  |  |  |  | rs4294502 | *TTC18* | NON_SYNONYMOUS_CODING | **2.03E-11** | 6.82E-03 | **6.28E-09** | **5.58E-15** |
|  |  |  |  | rs6480692 | *TTC18* | SYNONYMOUS_CODING | **1.93E-10** | 4.40E-04 | **4.59E-10** | **8.15E-26** |
|  |  |  |  | rs16930547 | *ANXA7* | INTRONIC | **1.33E-11** | 1.90E-04 | **2.48E-10** | **4.99E-19** |
|  |  |  |  | rs3763679 | *PPP3CB* | INTRONIC | 5.95E-04 | 1.25E-01 | 3.83E-03 | **2.02E-08** |
| 13 | 12 | 8.8 | - | rs12307610 | *RIMKLB* | INTRONIC | **1.61E-14** | 4.08E-03 | **1.43E-09** | NA |
| 14 | 15 | 62.5 | - | rs7181518 | *TRIP4* | INTRONIC | **2.29E-08** | 1.98E-01 | 1.76E-04 | 2.80E-07 |
| 15 | 16 | 49.2 | - | rs882671 | *NKD1* | INTRONIC | 1.38E-01 | 8.45E-02 | 2.66E-02 | **9.66E-09** |

[**Table S4**.](#_top) **Prior distributions, posterior distributions and accuracy estimates of the ABC**

The posterior distributions and accuracy estimates correspond to the most probable MB-Y model and were scaled according to the split time of Yoruba and Pygmy ancestors of 3,000 generations (60,000 years, 20 years/generation time). The posterior values for effective population sizes are shown in chromosome units and split time in generations. Median, mean, 95% of confidence intervals of the accuracy distances computed over 1,000 replicates are given, as well as the percentage of simulations below distance of 0.25. The performance of the ABC method in the MB-Y model was tested by means of simulating data from the prior distributions and then computing the distance between the ABC estimates and the real value of each parameter as: absolute*(real-estimated)/real. For 1,000 replicates the mean, median, 95% CI of the distances and the proportion of simulations below distance of 0.25 were computed.

|  |  | **Posterior distributions** | | | | **Accuracy distances** | | | | |
| --- | --- | --- | --- | --- | --- | --- | --- | --- | --- | --- |
| **Parameter** | **Prior distributions** | **Median** | **Mean** | **2.5%** | **97.5%** | **Median** | **Mean** | **2.5%** | **97.5%** | **<0.25** |
| N_e_ Mbuti | Uniform (500,5000) | 11295 | 16062 | 1393.44 | 62625.18 | 1.30 | 0.56 | 0.05 | 4.83 | 23.5 |
| N_e_ Biaka | Uniform (500,5000) | 13254 | 17736 | 1585.58 | 59722.75 | 1.30 | 0.60 | 0.06 | 4.56 | 19.5 |
| N_e_ Yoruba | Uniform (500,5000) | 12294 | 16059 | 1257.03 | 51919.96 | 1.24 | 0.54 | 0.04 | 4.62 | 23.1 |
| Migration Biaka_Mbuti | Uniform (0,0.0005) | 0.00089 | 0.00138 | 0.00007 | 0.00591 | 4.41 | 0.75 | 0.07 | 14.05 | 17.7 |
| Migration Yoruba_Mbuti | Uniform (0,0.0005) | 0.00081 | 0.00134 | 0.00011 | 0.00512 | 2.50 | 0.56 | 0.06 | 6.36 | 22.2 |
| Migration Yoruba_Biaka | Uniform (0,0.0005) | 0.00089 | 0.00144 | 0.00011 | 0.00567 | 2.19 | 0.65 | 0.07 | 6.66 | 18.5 |
| Time Split Biaka_Mbuti | Uniform (time first split, 15000) | 1094.1 | 1189.2 | 311.65 | 2504.21 | 0.76 | 0.48 | 0.05 | 2.60 | 26.9 |
| Ancestral N_e_ Biaka_Mbuti | Uniform (500,5000) | 11802 | 16191 | 1412.75 | 56963.16 | 1.18 | 0.57 | 0.06 | 4.18 | 22.7 |
| Ancestral N_e_ Yoruba_Pygmies | Uniform (500,5000) | 7941 | 14013 | 654.83 | 63202.05 | 2.08 | 0.71 | 0.09 | 8.97 | 17.7 |
| Pmean | Uniform(0,0.5) | 0.7071 | 1.3281 | 0.016 | 6.32 | 12.08 | 0.78 | 0.10 | 13.54 | 13.8 |
| mutationMean | LogUniform(1.0/10000.0, 5.0/1000.0) | 0.00143 | 0.00694 | 0.00006 | 0.04579 | 4.00 | 0.83 | 0.09 | 16.73 | 14.8 |

[**Table S5**](#_top)**. List of 400 STRs used for the ABC analysis.**

| NA-D8S-2 | D20S481 | D11S1986 | GATA31H11P_5 | GATA22H02_12 |
| --- | --- | --- | --- | --- |
| D17S1298 | D1S1660 | D8S1113 | AAAT111_5 | GATA7F09_12 |
| NA-D7S-1 | D11S1981 | D17S1294 | ATAG078P_5 | GATA91H01_12 |
| D4S1625 | D17S1290 | D10S2327 | ATAG022_5 | GATA167C12_12 |
| D1S1728 | NA-D12S-1 | D6S1017 | AGAT030P_5 | AGAT084_12 |
| D10S2470 | D3S1764 | D19S246 | GATA141B10M_5 | GGAT2G06M_12 |
| D11S4463 | D2S1334 | D19S245 | TAGA010_5 | GGAA19H02_12 |
| D7S3046 | D2S434 | D19S254 | GATA142H05P_5 | GGAA22C05_12 |
| D7S3047 | D12S395 | NA-D12S-2 | GATA51A07P_5 | GATA5H03_12 |
| D5S1456 | D12S372 | F13A1-D6S | GATA12G02_5 | GATA86B09P_13 |
| D21S1432 | NA-D15S-1 | TPO-D2S | AGAT122_5 | AGAT110P_13 |
| D6S2410 | D1S1609 | D20S164 | GATA73D11P_5 | GATA6B07_13 |
| D4S1644 | D15S643 | D11S1304 | AGAT130_5 | GATA73A05_13 |
| D2S1360 | D13S796 | D20S451 | GATA51D11P_5 | GATA137B09_13 |
| NA-D1S-2 | D20S482 | D12S297 | GATA139B09P_5 | GATA100E02P_13 |
| D3S4529 | D2S1384 | D6S942 | AGAT126_5 | AGAT113Z_13 |
| D1S3721 | D19S433 | D8S1048 | AAAT072_5 | TCTA023P_14 |
| D11S2363 | D15S659 | D8S373 | TAAA014P_6 | AGAT116P_14 |
| D2S427 | D12S1064 | D7S821 | GATA29C09P_6 | GATA90G11M_14 |
| D18S535 | D11S2365 | D6S1051 | GATA11B08P_6 | ATAC026P_14 |
| NA-D1S-3 | D10S1239 | D5S1505 | GATA112F02P_6 | GATA91G06_14 |
| D5S2845 | D11S4464 | D4S2623 | GATA30A08M_6 | GATA51F04P_14 |
| D7S3051 | D9S934 | GATA194H05Z_1 | GATA136F05P_6 | GATA045_14 |
| D7S817 | D2S1391 | GGAA23C07_1 | GATA161A04P_6 | ATGG002_14 |
| D5S2849 | D19S714 | MFD424-TTTA003_1 | TATC050ZM_6 | GATA143C02_15 |
| D8S2324 | D3S2460 | GATA23G09_1 | ATGA020_6 | GATA153F11_15 |
| NA-D8S-1 | D6S1056 | TTTA063P_1 | GATA61G06_7 | AATA053_15 |
| D15S1507 | D1S551 | GATA2B02Z_1 | TATC010P_7 | GATA63B12P_15 |
| D6S2439 | D11S1392 | AGAT143_1 | TATT019_7 | AGAT073P_15 |
| D14S1434 | D18S851 | GGAA20F08_1 | GATA137A12M_7 | GATA22F01_15 |
| D18S1371 | D5S820 | GATA13C08M_1 | GATA4E04_7 | TTTA028_16 |
| NA-D18S-1 | NA-D4S-1 | TATC028_1 | GATA21D12P_7 | GATA5H07M_16 |
| D2S2968 | D10S1423 | AGAT118_1 | GATA87D11_7 | GATA86C08P_16 |
| D18S1376 | D4S2632 | GATA51H01_1 | ATAC037P_7 | TTAT023Z_16 |
| D17S2196 | D2S1780 | AATA011_1 | TTTA001M_7 | CATA002Z_16 |
| NA-D9S-1 | D1S2134 | GATA28H06_2 | AGAT133_7 | GATA143D05_16 |
| D21S2055 | D7S2204 | GATA056_2 | GATA145G10M_7 | GATA151C03P_16 |
| D7S3070 | D10S1426 | AGAT117_2 | GATA63F08P_7 | GATA140E03_16 |
| NA-D14S-1 | D15S816 | GATA194B06P_2 | AGAT049P_7 | TCTA026_16 |
| NA-D22S-1 | D12S1294 | GATA10H05_2 | TATG002P_7 | GATG013M_16 |
| D22S689 | D14S742 | GATA130A05M_2 | MFD442-GTTT002_7 | GATA158H04_17 |
| D1S1594 | D5S1470 | GATA70F12M_2 | ATAA009_8 | AGAT132_17 |
| D19S586 | D9S301 | GATA148G10P_2 | TTCA004P_8 | GATA64B04P_17 |
| D2S1363 | D18S1364 | GATA139A04P_2 | GATA25C10M_8 | GATA10H07P_17 |
| D11S1998 | D3S3039 | TAGA002M_2 | TATC012_8 | GATA169F02_17 |
| D7S1799 | D8S1179 | GATA8H05_2 | ATAA018P_8 | GGAA19G04_17 |
| D11S1999 | D6S1277 | AGAT093_2 | AATA019_8 | AAT245_17 |
| D7S3056 | D9S1120 | GATA63F01_2 | GATA156H01M_8 | GATA31B11_17 |
| D17S1299 | D16S2624 | GATA43F06_2 | ATAG042_8 | GATA63G01_17 |
| D8S1132 | D3S3045 | GATA126A06M_2 | GATA3H11_8 | TTCA006M_17 |
| D1S1596 | D10S1430 | GATA91D12M_2 | AAAT121P_8 | AGAT060_18 |
| D2S1328 | D12S1300 | GATA194A05M_2 | GATA060_8 | AGAT127_18 |
| D3S2432 | D15S818 | GATA149B10M_2 | GATA175H06M_9 | GATA062_18 |
| D1S1597 | D2S1788 | GATA23A02_2 | AGAT142P_9 | GATA85D10_18 |
| D4S2368 | D13S894 | AGAT021_2 | TCTA020_9 | GATA173A03_18 |
| D17S1301 | D9S1121 | MFD433-AGAT010_3 | GATA165A11M_9 | ATAG089P_18 |
| D4S3248 | D10S1432 | ATCT053P_3 | GATA5E06P_9 | GATA157H01_18 |
| D6S1959 | D2S1790 | GATA178C11M_3 | AGAT140P_9 | AGAT138P_18 |
| D1S3669 | D9S1122 | GATA146B10_3 | GATA22H04M_9 | GATA146H09_19 |
| D19S589 | D4S1629 | GATA87B02_3 | GGAT3G09M_9 | GGAA21A04_19 |
| D4S1647 | D3S1768 | AGAT128_3 | AAAT126_9 | GATA156F11_19 |
| D5S816 | D17S974 | GGAT2G03_3 | GATA27Z_9 | TTTA075P_19 |
| D14S606 | D3S1746 | GATA146D07_3 | GATA65D11_9 | AGAT136M_20 |
| D14S1280 | D8S1110 | TTTA033_3 | TCTA017M_9 | ATTC013_20 |
| D6S1009 | D11S2371 | GATA152F04M_3 | CTAT016_9 | GATA81E09_20 |
| D12S2078 | D12S1301 | GATA92B06P_3 | ATAG053P_10 | AGAT139P_20 |
| D3S1744 | D14S587 | TTTA040_3 | ATCC001_10 | GATA142C02M_20 |
| D3S1763 | D11S1984 | CTAT012_3 | ATGT009P_10 | GATA65E01_20 |
| D5S1462 | D4S2431 | MFD427-AAAT028_3 | ATAG055_10 | GATA90E02_20 |
| D8S1136 | D8S1477 | ATCT018_4 | GATA179E06P_10 | ATCT035_20 |
| D7S1802 | D9S938 | GATA29_4 | TCTA021ZM_10 | TATT031_20 |
| D20S478 | D13S895 | GAAT1F09P_4 | GATA196C10P_10 | TATC057_21 |
| D16S764 | D1S1677 | TAGA006_4 | GATA134F03P_10 | AGAT057_21 |
| D4S2417 | D10S1248 | GATA30B11_4 | GATA88F08P_10 | AGAT120_22 |
| D7S1804 | D13S1493 | GATA135C03M_4 | TACA003_10 | ATTT019M_22 |
| D14S608 | D7S1808 | GATA150B10_4 | ATGT006Z_10 | GATA6F05P_22 |
| D13S793 | D16S753 | GATA138G03_4 | GATA29B11_11 | TCAT006ZP_22 |
| D19S591 | D14S588 | TAGA049_4 | TCTA025_11 | TTAT020P_22 |
| D20S480 | D3S2403 | AATA045_4 | GATA35_11 | GATA030P_22 |
| D11S2006 | D7S3061 | AGAT099P_5 | GATA101G01_12 | TCTA015M_22 |

**Supplemental figures.**

[**Figure S1.**](#_top) **Validation of the Conditional Informativeness statistic.**

**A.** Results for covariation of lactase persistence phenotype in Europeans. Candidate region for the lactase persistence phenotype in Europeans (TSI and CEU from HapMap 3), and French from the HGDP-CEPH Illumina 650Y data(Li et al. 2008)). Manhattan plot showing the results of the spatial autocorrelation in CI_n_ for the 22 autosomal chromosomes. The red line indicates the significance threshold after Bonferroni correction. The smaller plot in the right represents a zoom of the region above the significance threshold. Full diamonds represent significant SNPs and the big diamond corresponds to the SNP showing the smallest p-value (indicated). The recombination rates (HapMap Release 22) and genes (from UCSC, Build36 coordinates) in the region are also shown. The respective lactase persistence frequencies for each population (0.21, 0.85 and 0.68) were retrieved from (Itan et al. 2010).

**B.** The histogram shows the frequency of each of the possible orders for minor allele frequencies for the five populations considered in the CI_n_ analysis (1: Mbuti, 2: Biaka, 3: Yoruba, 4: Luhya, 5: Maasai). We named scenario 1 those simulations in which the phenotype was generated based on causal SNPs that followed the phenotypic cline which coincides with the two most common ones (asterisked in black, Mbuti ≤ Biaka < Yoruba/Luhya < Maasai). Scenario 2, the causal SNPs followed the same population order than in scenario 1 but additionally showed a minimum minor allele frequency difference between Maasai and Mbuti of 0.37. For the third and fourth scenarios, we considered the causal SNPs obtained in the first and second scenarios, but switched the labels so that the causal allele frequencies and the subsequent phenotypes followed a rare cline in the genome (asterisked in blue).

**
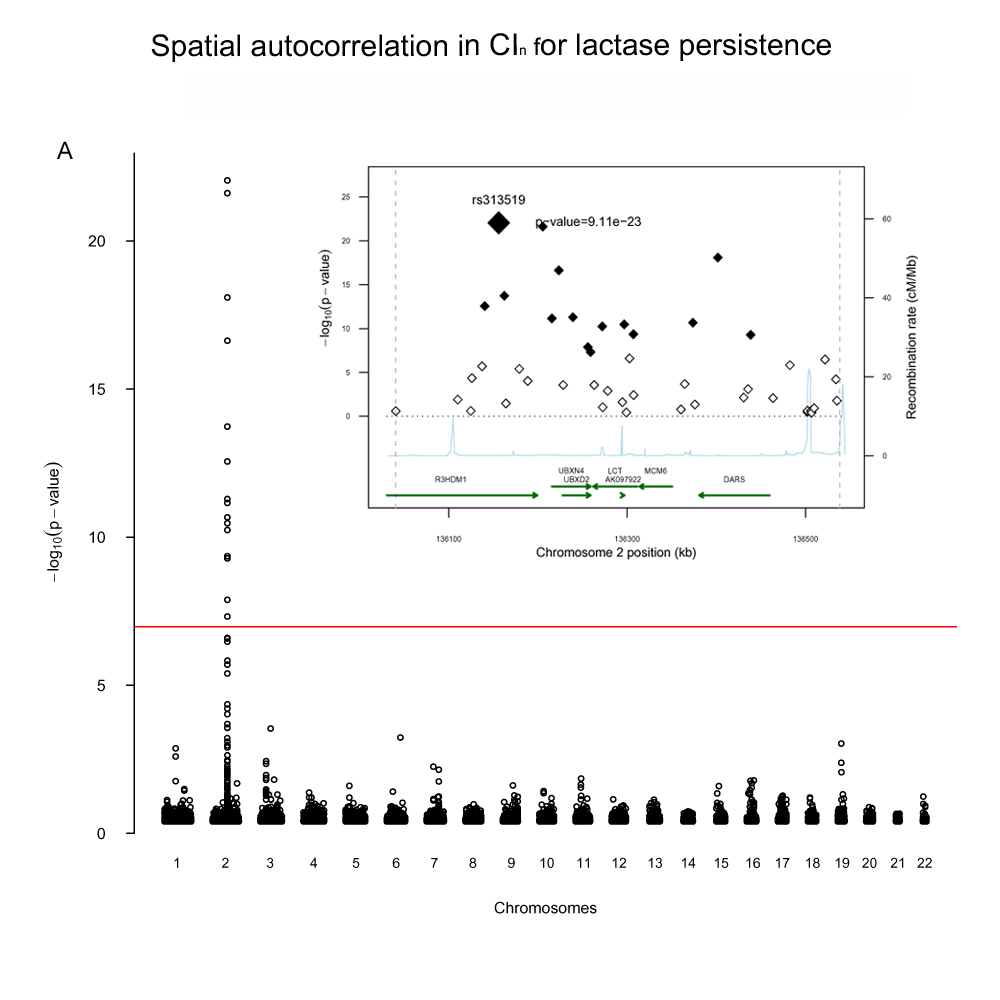
**

[**Figure S2.**](#_top) **Demographic simulations.**

**A.** Models tested by ABC. The models represent the three possible topologies: i) the MB-Y model, in which the Pygmy populations share a more recent common ancestry than with Yoruba ii) the BY-M model, representing the most ancient split between Mbuti and the common ancestor of Yoruba and Biaka and iii) the MY-B model, involving an ancient divergence of Biaka and the ancestor of Yoruba and Mbuti. All three models allowed for migration between all three populations and considered constant population sizes.

**B-D.** Features of the simulated dataset SNPs. **B.** Pairwise distances (kb) between 1,000 SNP pairs in the Illumina French dataset and in the simulated European dataset used for analysis (Cosi after ascertainment bias correction). C. MAF distribution in the Illumina French dataset, in the original simulated European Cosi dataset and in the final dataset used for analysis (Cosi after ascertainment bias correction). **D**. r^2^ between 500 pairs of SNPs in the simulated European

Cosi dataset after ascertainment bias correction and in the Illumina French dataset.

[**Figure S3.**](#_top) **Candidate regions for the Pygmy phenotype**.

In each plot, the upper plot shows the –log_10_(p-values) for the four analyses performed in the area limited by dashed grey lines in the lower plot. The lower plot shows the log_10_(p-values) of the analysis with highest significance (diamond color according to the legend) with the recombination rates (HapMap Release 22) and genes in the region (from UCSC, Build36 coordinates). Full diamonds are significant SNPs after Bonferroni correction and the big diamond corresponds to the SNP showing the smallest p-value (indicated**)**.

The haplotype plots (pages 23-26) for the African populations from HGDP Selection Browser (Coop et al. 2009; Pickrell et al. 2009; Pritchard et al. 2010). The figures show haplotype patterns in a genomic region, where each row in the plot is a haplotype, and each column is a SNP. The rows are coloured so that all haplotypes of the same colour are identical. The algorithm for generating the plots is described in Conrad et al. (2006).

[**Figure S4.**](#_top) **Plots of the statistics for the observed and simulated datasets.**

The simulated dataset consists on 10,000 SNPs drawn from 1,000 demographic scenarios. Grey points represent the mean value of the statistic for all 1,000 demographies, the gray lines the 2.5 and 97.5 % quantiles and the grey dashed lines the maximum and minimum values observed in all simulations. The observed dataset consists on 1,000 samplings of 10,000 SNPs from Illumina dataset used in this study. Blue points indicate the mean value of the statistics for the 1,000 samplings, the blue lines the 2.5 and 97.5 % quantiles and the blue dashed lines the maximum and minimum values. Red lines delimit the significance threshold applied in the study (after Bonferroni correction) and black lines point out the maximum value observed in the simulations for the LMI-PI_n_ statistic.

[**Figure S5.**](#_top) **Results for the Ingenuity Pathway Analysis.**

The figure shows the results of the comparison of the 33 genes detected by being strongly differentiated in Pygmies, PI_n_ **(A)** and the 24 genes that strongly covaried with height CI_n_ **(B)** to the canonical pathways in the Ingenuity Pathways Knowledge Base repository. The p-values correspond to the Fisher’s exact test of the association between the genes in the dataset and the canonical pathway is explained by chance alone.

[**Supplemental References**](#_top)

Altshuler DM, Gibbs RA, Peltonen L, Altshuler DM, Gibbs RA, Peltonen L, Dermitzakis E, Schaffner SF, Yu F, Peltonen L, Dermitzakis E, Bonnen PE, Altshuler DM, Gibbs RA, de Bakker PI, Deloukas P, Gabriel SB, Gwilliam R, Hunt S, Inouye M, Jia X, Palotie A, Parkin M, Whittaker P, Yu F, Chang K, Hawes A, Lewis LR, Ren Y, Wheeler D, Gibbs RA, Muzny DM, Barnes C, Darvishi K, Hurles M, Korn JM, Kristiansson K, Lee C, McCarrol SA, Nemesh J, Dermitzakis E, Keinan A, Montgomery SB, Pollack S, Price AL, Soranzo N, Bonnen PE, Gibbs RA, Gonzaga-Jauregui C, Keinan A, Price AL, Yu F, Anttila V, Brodeur W, Daly MJ, Leslie S, McVean G, Moutsianas L, Nguyen H, Schaffner SF, Zhang Q, Ghori MJ, McGinnis R, McLaren W, Pollack S, Price AL, Schaffner SF, Takeuchi F, Grossman SR, Shlyakhter I, Hostetter EB, Sabeti PC, Adebamowo CA, Foster MW, Gordon DR, Licinio J, Manca MC, Marshall PA, Matsuda I, Ngare D, Wang VO, Reddy D, Rotimi CN, Royal CD, Sharp RR, Zeng C, Brooks LD, McEwen JE (2010) Integrating common and rare genetic variation in diverse human populations. Nature 467: 52-8

Batini C, Lopes J, Behar DM, Calafell F, Jorde LB, van der Veen L, Quintana-Murci L, Spedini G, Destro-Bisol G, Comas D (2011) Insights into the demographic history of African Pygmies from complete mitochondrial genomes. Mol Biol Evol 28: 1099-110

Beaumont MA, Zhang W, Balding DJ (2002) Approximate Bayesian computation in population genetics. Genetics 162: 2025-35

Campbell MC, Tishkoff SA (2010) The evolution of human genetic and phenotypic variation in Africa. Curr Biol 20: R166-73

Cavalli-Sforza LL (1986) African Pygmies. Harcourt Brace Jovanovich, Orlando, Florida

Clark AG, Hubisz MJ, Bustamante CD, Williamson SH, Nielsen R (2005) Ascertainment bias in studies of human genome-wide polymorphism. Genome Res 15: 1496-502

Conrad DF, Jakobsson M, Coop G, Wen X, Wall JD, Rosenberg NA, Pritchard JK (2006) A worldwide survey of haplotype variation and linkage disequilibrium in the human genome. Nat Genet 38: 1251-60

Coop G, Pickrell JK, Novembre J, Kudaravalli S, Li J, Absher D, Myers RM, Cavalli-Sforza LL, Feldman MW, Pritchard JK (2009) The role of geography in human adaptation. PLoS Genet 5: e1000500

Cover TM, Thomas JA (1991) Elements of Information Theory. Wiley-Interscience, New York

Christensen DL, Eis J, Hansen AW, Larsson MW, Mwaniki DL, Kilonzo B, Tetens I, Boit MK, Kaduka L, Borch-Johnsen K, Friis H (2008) Obesity and regional fat distribution in Kenyan populations: impact of ethnicity and urbanization. Ann Hum Biol 35: 232-49

de Gruijter JM, Lao O, Vermeulen M, Xue Y, Woodwark C, Gillson CJ, Coffey AJ, Ayub Q, Mehdi SQ, Kayser M, Tyler-Smith C (2011) Contrasting signals of positive selection in genes involved in human skin color variation from tests based on SNP scans and resequencing. Investig Genet 2: 24

Dubuisson M, Jain A A modified Hausdorff distance for object matching Proceedings of the 12th IAPR International Conference on Pattern Recognition, Jerusalem, Israel 1994. IEEE Computer Society Press, pp 566 - 568

Eltis D (1982) Nutritional Trends in Africa and the Americas: heights of Africans, 1819-1839. The Journal of Interdisciplinary History XII: 453-475

Itan Y, Jones BL, Ingram CJ, Swallow DM, Thomas MG (2010) A worldwide correlation of lactase persistence phenotype and genotypes. BMC Evol Biol 10: 36

Kayser M, Brauer S, Stoneking M (2003) A genome scan to detect candidate regions influenced by local natural selection in human populations. Mol Biol Evol 20: 893-900

Laval G, Excoffier L (2004) SIMCOAL 2.0: a program to simulate genomic diversity over large recombining regions in a subdivided population with a complex history. Bioinformatics 20: 2485-7

Li JZ, Absher DM, Tang H, Southwick AM, Casto AM, Ramachandran S, Cann HM, Barsh GS, Feldman M, Cavalli-Sforza LL, Myers RM (2008) Worldwide human relationships inferred from genome-wide patterns of variation. Science 319: 1100-4

McVean G (2009) A genealogical interpretation of principal components analysis. PLoS Genet 5: e1000686

Patin E, Laval G, Barreiro LB, Salas A, Semino O, Santachiara-Benerecetti S, Kidd KK, Kidd JR, Van der Veen L, Hombert JM, Gessain A, Froment A, Bahuchet S, Heyer E, Quintana-Murci L (2009) Inferring the demographic history of African farmers and pygmy hunter-gatherers using a multilocus resequencing data set. PLoS Genet 5: e1000448

Pickrell JK, Coop G, Novembre J, Kudaravalli S, Li JZ, Absher D, Srinivasan BS, Barsh GS, Myers RM, Feldman MW, Pritchard JK (2009) Signals of recent positive selection in a worldwide sample of human populations. Genome Res 19: 826-37

Pritchard JK, Pickrell JK, Coop G (2010) The genetics of human adaptation: hard sweeps, soft sweeps, and polygenic adaptation. Curr Biol 20: R208-15

Rosenberg NA (2006) Standardized subsets of the HGDP-CEPH Human Genome Diversity Cell Line Panel, accounting for atypical and duplicated samples and pairs of close relatives. Ann Hum Genet 70: 841-7

Rosenberg NA, Li LM, Ward R, Pritchard JK (2003) Informativeness of genetic markers for inference of ancestry. Am J Hum Genet 73: 1402-22

Rosenberg NA, Mahajan S, Ramachandran S, Zhao C, Pritchard JK, Feldman MW (2005) Clines, clusters, and the effect of study design on the inference of human population structure. PLoS Genet 1: e70

Schaffner SF, Foo C, Gabriel S, Reich D, Daly MJ, Altshuler D (2005) Calibrating a coalescent simulation of human genome sequence variation. Genome Res 15: 1576-83

Storz JF, Payseur BA, Nachman MW (2004) Genome scans of DNA variability in humans reveal evidence for selective sweeps outside of Africa. Mol Biol Evol 21: 1800-11

Verdu P, Austerlitz F, Estoup A, Vitalis R, Georges M, Thery S, Froment A, Le Bomin S, Gessain A, Hombert JM, Van der Veen L, Quintana-Murci L, Bahuchet S, Heyer E (2009) Origins and genetic diversity of pygmy hunter-gatherers from Western Central Africa. Curr Biol 19: 312-8

Wang C, Szpiech ZA, Degnan JH, Jakobsson M, Pemberton TJ, Hardy JA, Singleton AB, Rosenberg NA (2010) Comparing spatial maps of human population-genetic variation using Procrustes analysis. Stat Appl Genet Mol Biol 9: Article 13

Wegmann D, Leuenberger C, Excoffier L (2009) Efficient approximate Bayesian computation coupled with Markov chain Monte Carlo without likelihood. Genetics 182: 1207-18
